# Unveiling the Electronic Structure of the Bi(+1)/Bi(+3) Redox Couple on NCN and NNN Pincer Complexes

**DOI:** 10.1021/acs.inorgchem.1c02252

**Published:** 2021-11-12

**Authors:** Martí Gimferrer, Sergi Danés, Diego M. Andrada, Pedro Salvador

**Affiliations:** † Institut de Química Computacional i Catàlisi and Departament de Química, Universitat de Girona, Maria Aurèlia Capmany 69, 17003 Girona, Catalonia, Spain; ‡ Faculty of Natural Sciences and Technology, Department of Chemistry, Saarland University, 66123 Saarbrücken, Federal Republic of Germany

## Abstract

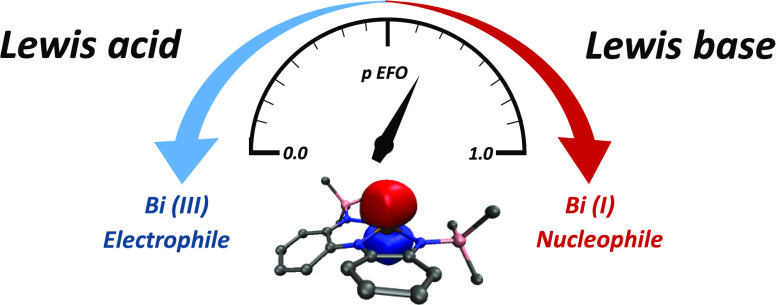

Low-valent group 15 compounds stabilized by pincer ligands have gained particular interest, given their direct access to fine-tune their reactivity by the coordination pattern. Recently, bismuth has been employed in a variety of catalytic transformations by taking advantage of the (+1/+3) redox couple. In this work, we present a detailed quantum–chemical study on the electronic structure of bismuth pincer complexes from two different families, namely, bis(ketimine)phenyl (NCN) and triamide bismuthinidene (NNN). The use of the so-called effective oxidation state analysis allows the unambiguous assignation of the bismuth oxidation state. In contrast to previous studies, our calculations suggest a Bi(+1) assignation for NCN pincer ligands, while Bi(+3) character is found for NNN pincer complexes. Notably, regardless of its oxidation state, the central bismuth atom disposes of up to two lone pairs for coordinating Lewis acids, as indicated by very high first and second proton affinity values. Besides, the Bi–NNN systems can also accommodate two Lewis base ligands, indicating also ambiphilic behavior. The effective fragment orbital analysis of Bi and the ligand allows monitoring of the intricate electron flow of these processes, revealing the noninnocent nature of the NNN ligand, in contrast with the NCN one. By the dissection of the electron density into effective fragment orbitals, we are able to quantify and rationalize the Lewis base/acid character.

## Abstract 2

Effective oxidation state analysis sheds light on the electronic structure of chemical systems. The oxidation state of bismuthinidene pincer complexes can be assigned as Bi(+1) or Bi(+3) depending on the nature of the ligands. Despite this assignation, the reactivity pattern as Lewis base or acid is similar. The occupation of the effective fragment orbitals gives a straightforward method to quantify the reactivity.

## Introduction

In recent years, there has been an increasing interest in using heavier main group elements as a potential replacement of transition metals (TMs) in catalytic reactions.^1−3^ The work on heavier group 15 elements, “pnictogen(Pn)-based” species P, As, Sb, and Bi, has showcased their capability to participate as catalysts in a number of reaction transformations.^4−8^

It has been recognized that the activity sharply depends on the nature of the ligand and the pnictogen center since special combinations allow to fine-tune the geometry and the oxidation state of the central pnictogen atom. Thus, a number of complexes with different rigidities, steric protection, and pnictogen centers have been experimentally accomplished.^9−11^ Bismuth has brought plenty of possibilities given its ability to adopt all oxidized and reduced states from +5 to −3.^12,13^ Bi-based complexes can act as catalysts in a wide variety of chemical reactions, namely, in the activation of challenging bonds,^6,8^ CO_2_ fixation,^14,15^ or as precursors in materials science,^16,17^ among others. For a recent review on bismuth catalysis, see ref (18).

**Figure 1:**
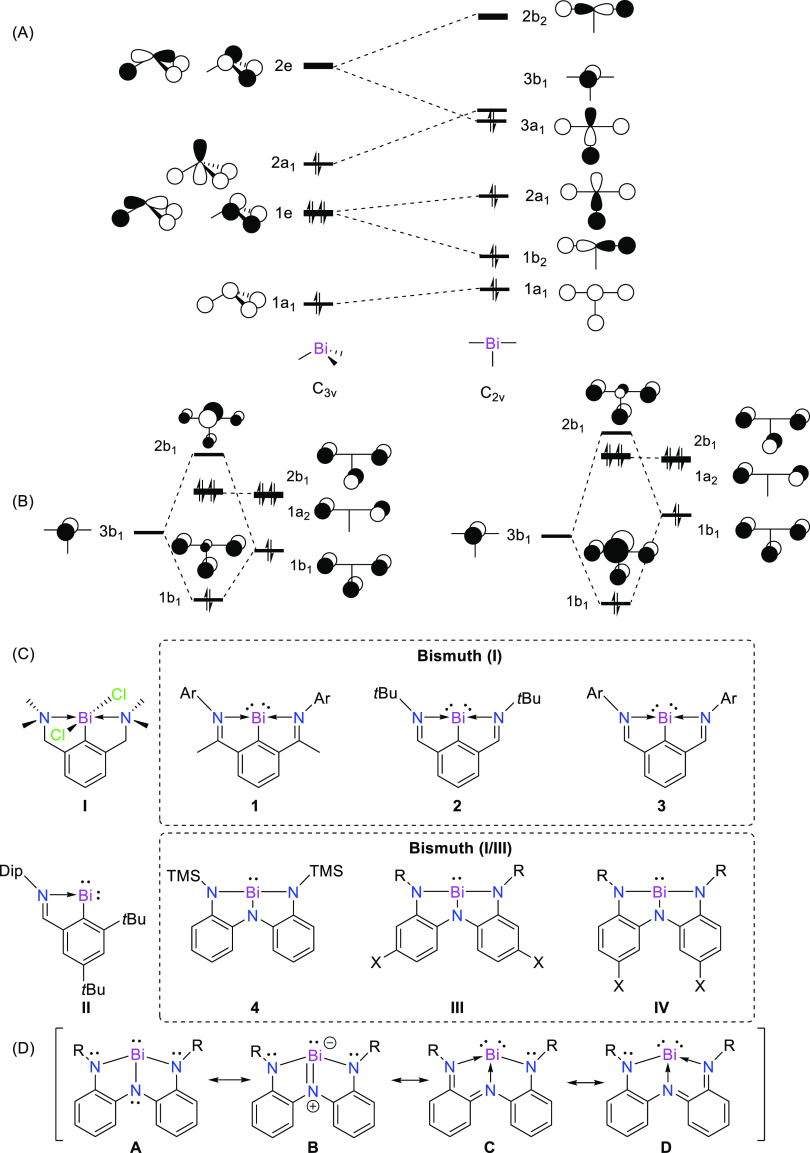
(A) Qualitative frontier molecular orbital diagram of BiH_3_ in *C*_3v_ and *C*_2v_ symmetries. (B) Molecular orbital diagram of interaction between bismuth and the conjugated pincer; weak (left) and strong (right) π-donors. (C) Bi-based complexes: **I** (ref (22)), **II** (ref (23)) **1** Ar = 2,6-Me_2_C_6_H_3_ (ref (24)) **2**, **3** Ar = 4-Me_2_NC_6_H_4_ (refs (23, 25)), **4** (ref (26)), **III**, and **IV** (ref (27)). Dip = 1,3-diisopropylphenyl; TMS = trimethylsilyl; tBu = *tert*-butyl. (D) Possible resonance structures.

Efforts are justified as nontoxic bismuth has potential applications in medicinal chemistry, in contrast to its lighter congeners (P, As, and Sb).^19−21^ The utilization of tridentate rigid meridional pincer ligands has been the key to engineering the energetic levels of frontier orbitals, encompassing similar chemical bonding and reactivity patterns to transition metals and, in some cases, exhibiting unprecedented reactivity.^1^ The pyramidal *C*_3v_ coordination mode has a lone pair in an *a*_1_ orbital, while the *e* degenerated orbitals are located high in energy, resulting in typical Lewis base behavior (Figure 1A).

Pincer ligands enforce a *C*_2v_ coordination mode (T-shape), where the lone pair becomes an empty p-orbital (*b*_1_), and one of the *e* antibonding orbitals reduces its energy, becoming *a*_1_ lone pair in the plane of the ligand. As a result, the HOMO–LUMO gap is considerably reduced, resembling the electronic situation of a transition metal. Such a bonding situation engages reactivity as a Lewis base or acid.

Notably, the use of pincer ligands with π-conjugated systems gives another channel to tailor the reactivity via conjugation with the empty p-orbital interaction (Figure 1B). The p-orbital (*b*_1_) of the bismuth atom can interact with *b*_1_-orbitals of the pincer ligand on the π-system. The resulting π-bonding orbital can be located either at the bismuth or at the ligand, depending on the relative energy level of the constituting fragments, leading to an oxidation state of +1 or +3, respectively.

Soran et al. described the synthesis of organobismuth(+3) dihalide containing (NCN)-pincer ligand **I**.^22^ The complexes presented a T-shaped CBiCl_2_ core stabilized by two intramolecular dative N → Bi bonds. After that, Šimon et al. characterized the first examples of a monomeric bismuthinidene **1**.^24^ The use of 2,6-bis(ketimine)phenyl ligand ensured steric protection of the orbitals at central bismuth. Similar ligands were later used by Vránová et al. to access **2** and **3** via reduction of the corresponding chelated bismuth chlorides.^23,25^ They demonstrated that the reduction outcomes are influenced by the strength of the N → Bi interaction. This led to the rational design of unprecedented two-coordinated bismuthinidene **II**.^23^

The presence of the bismuth lone pair has been proven by the ability to coordinate various transition-metal carbonyl moieties.^24^ Recently, Cornella et al. demonstrated the capacity of bismuth compounds to be engaged in catalytic redox transformations by making use of the oxidation states +1 and +3. Thus, complex **2** resulted useful for the transfer hydrogenation of azoarenes and nitroarenes with ammonia-borane as a transfer agent.^5^ Mechanistic investigations suggested a Bi(+3) hydride as the key intermediate. The same group showed that N_2_O activation is facilitated by low-valent bismuth complexes through the formation of a Bi(+3) = O intermediate.^28^

**Scheme 1:**
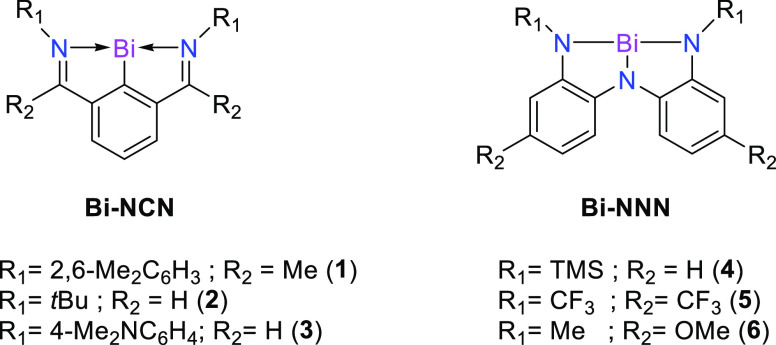
Molecular Systems of Bismuth Pincer Complexes Considered in This Study

The first example of a planar geometry for bismuth triamides **4** has been recently described by Kindervater et al.^26^ The term “redox-confused” was coined for this compound, as it has significant Bi(+1) character but also exhibits reactivity similar to Bi(+3) electrophiles. The coordination of either pyridine *N*-oxide or W(CO)_5_ revealed either a vacant or a filled 6p_*z*_-orbital at the Bi atom. Noteworthily, the assignation of **4** as a Bi(+1) species was based on previous NCN-coordinated compounds. Nonetheless, its preparation uses a Bi(+3) precursor to yield **4** without external reduction agents. This chemical behavior points toward rather ambiguous oxidation state (OS) labeling. Marczenko et al. studied the periodic trends in the structure, bonding, and reactivity of E-NNN species, where E = P, As, Sb, and Bi(**4**).^29^ Their experimental and computational findings suggested a major tendency to adopt planar geometries the heavier the central atom (i.e. going down the group), which carries an evident increase in the acidity. In a subsequent study, Marczenko et al.^27^ computationally explored the fine tuning of the Lewis acidity character by substitution on the aryl ring. Introducing electron-withdrawing groups such as −CF_3_ (**5**) induced stronger Lewis acid character, while electron-donating groups such as −OCH_3_ (**6**) lead to lower acidity, compared to **4** (see Scheme 1).

A redox couple involving closed-shell species in combination with the absence of unpaired electrons/spin density makes the oxidation state (OS) assignation particularly difficult with traditional approaches.^30,31^ The OS is inherently connected to the electron distribution around the atom. Several schemes based on computational methods have been recently developed to assist in the task of OS assignation in dubious cases. Rather than relying on average quantities such as partial atomic charges or spin populations, these schemes assign individual (or pairs of) electrons to the atoms or fragments/ligands of the compound. Many of these approaches take advantage of the use of localized orbitals.^32−36^ We have recently developed an automated method so-called effective oxidation state (EOS) analysis.^36^ This method is based on Mayer’s spin-resolved effective fragment orbitals (EFOs)^37,38^ and their occupations (λ) to perform the OS assignation. The EFOs are the eigenvectors of the net fragment overlap matrix, and the corresponding eigenvalues represent the occupation numbers. Thus, the EFOs are the orbitals of the fragment’s net density and, as such, they are normalized within the fragment boundary. They are obtained for each atom/fragment separately. In EOS assignation, rather than rounding the occupation to the nearest integer, the total number of α and β electrons are assigned to those EFOs with higher occupation numbers. Thus, no occupation threshold is introduced. This procedure leads to an effective configuration of each atom or fragment and hence its OS. The difference in the occupation between the last occupied (λ_LO_) and first unoccupied (λ_FU_) EFO indicates to which extent the electron distribution can be pictured as a discrete ionic model. In addition, a reliability index, *R* (%) = min (*R*_α_, *R*_β_), of the OS distribution can be defined for each spin case σ (α or β) as
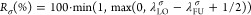
1The OS assignment is considered as undisputable (*R* (%) = 100) when the difference in occupation of the frontier EFOs exceeds half-electron. The worst-case scenario occurs when two or more frontier EFOs from different fragments present the same occupation. In this case, two different equally plausible OS distributions would be present with *R* (%) = 50. Experience indicates that undisputable OS assignments are usually obtained for textbook examples of TM compounds, while *R* (%) values of around 65–70 are expected for systems with more intricate electronic structures.^39^ The presence of noninnocent or redox-active ligands such as nitrosyl may lead to close-call situations with *R* (%) < 60 between NO(+)/NO(−) due to the high covalent character of the sigma metal–nitrosyl bond.^40^ Similar high covalent character was also observed for the Ru–C bonds along the catalytic cycle of Ru-based olefin metathesis.^41^

EOS analysis has already been successfully applied to a wide variety of systems.^39,40,42−44^ Most of the systems studied so far involved transition-metal compounds, but the EOS method is of general applicability. Herein, we extend the EOS scope into main group chemistry using this tool to tackle the intriguing Bi(+1/+3) redox couple. The systems considered in this work include monomeric bis(ketimine)phenyl (Bi–NCN) and triamide bismuthinidene (Bi–NNN), given their rather challenging and ambiguous bonding picture. Thus, the description by different resonance structures (Figure 1D) may lead to either oxidation state +1 or +3, which can be reduced to the question: does bismuth possess one or two lone pairs? To gain insight into the electronic structure of these complexes, we examined the oxidation state involving a series of structural variations where the size of the flanking groups *R*_1_ is increased, and the electronic nature of the π-conjugated system is tuned by donor or electron-withdrawing groups. Beyond the mere assignation of a formal OS, the visualization of the frontier EFOs unambiguously shed light on the intricate electronic structure of these compounds. Besides, EOS analysis provides reliable and robust quantification of the Lewis acid/base character from ground-state properties, without recurring to intermediate states.

## Results and Discussion

**Figure 2:**
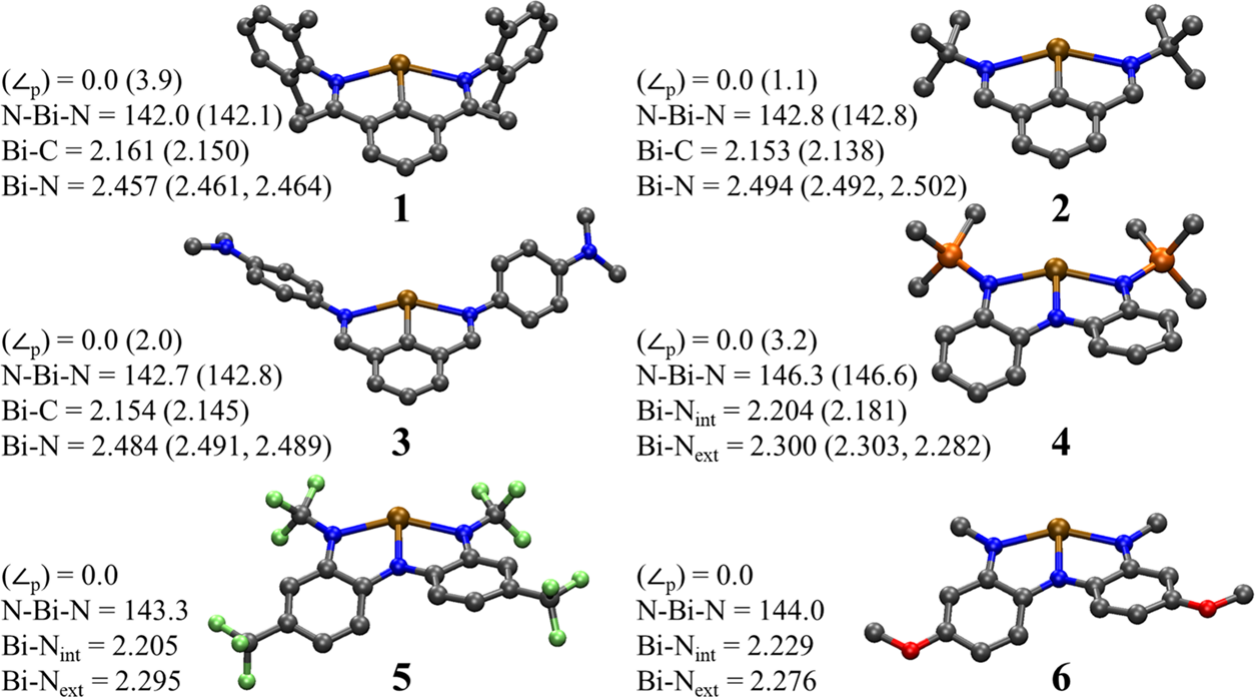
Optimized Bi-pincer complexes with selected bond distances (in Å) and bond angles (in deg) at B3LYP-D3(BJ)/def2-TZVPP, experimental data (in parentheses) from refs (23, 24) and (26). The pyramidalization angle (∠_p_) has been taken as the dihedral angle N–C–N–Bi and N–N–N–Bi. Hydrogen atoms were omitted for clarity.

Figure 2 shows the calculated optimized geometries at the B3LYP-D3(BJ)/def2-TZVPP level of theory for the studied bismuth complexes outlined in Scheme 1. The equilibrium geometries are in very good agreement with the experimental ones, when available, or with previous computational studies.^23,24^ NCN-based systems **1**–**3** present a planar central moiety with a general *C*_2v_ symmetry. The pyramidalization angles of bismuth (∠_p_), taken as the dihedral angles N–C–N–Bi, are 0.0° for all computed species, while the experimentally determined are lower than 4.0° (see Figure 2).

The computed Bi–C bond lengths slightly vary throughout the series, i.e., 2.161 Å (**1**), 2.153 Å (**2**), and 2.154 Å (**3**), lying within the expected bond length of a Bi–C (2.26 Å) single and Bi=C (2.08 Å) double bond.^45^ In the case of Bi–N, the bond lengths range from 2.457 Å (**1**) to 2.494 Å (**2**), which are longer than the expected bond length of a Bi–N (2.22 Å) single bond,^45^ pointing to a N → Bi donor–acceptor interaction as suggested elsewhere.^20^ The reported average experimental bond lengths are 2.150 Å (**1**), 2.138 Å (**2**), and 2.145 Å (**3**) for Bi–C and 2.463 Å (**1**), 2.496 Å (**2**), and 2.490 Å (**3**) for Bi–N. Although the crystal structures are not completely symmetric, there is a very good agreement with the computed ones.

The coordination of the NNN ligand in **4**–**6** is essentially planar, but the H···H repulsion between the aryl moieties induces a tilt of about 30°. This effect lowers the symmetry of the systems from *C*_2v_ to *C*_2_.^26^ The experimental average Bi–N_ext_ distance in **4** (2.292 Å) is in agreement with our DFT-optimized value of 2.300 Å. These values are also longer than the expected distance of a Bi–N single bond but shorter than in **1–3** complexes. Besides, the central Bi–N_int_ displays a shorter bond length (2.201/2.181 Å), suggesting a single bond with a weak double bond character. Such structural changes could imply a different oxidation state according to the ligand nature.

Thus, we have applied EOS analysis (see the Computational Details section for further technical details) to determine the oxidation state of bismuth. All calculations have been performed at the B3LYP-D3(BJ)/def2-TZVPP level of theory. First, since EOS had been mostly applied to TM systems, we have tested the method against a chemically diverse set of 19 Bi-based systems. The OS assignations are very clear in almost all cases (*R*% > 75) and in perfect agreement with the expected OS (see Table S7). The only significant exception is a dibismuthene species, for which the rather low *R* (%) = 58 value emerges from the essentially unpolarized covalent nature of the Bi–Bi bond. Table 1 gathers the predicted OS of systems **1**–**6**, where the fragments are the Bi atom and the pincer ligands. The occupations of the relevant EFOs for the Bi atom and the pincer ligand are also included, together with the reliability index *R* (%).

**Table 1:** Frontier EFO Occupations (in au) of Bi and Pincer Ligand (NCN or NNN) and the Assigned Oxidation States of **1**–**6** Complexes

	6s Bi	6p_*z*_ Bi	π L	OS Bi	OS L	*R* (%)
1	0.93	0.59	0.41	+1	–1	68.6
2	0.93	0.59	0.41	+1	–1	68.1
3	0.93	0.60	0.40	+1	–1	69.6
4	0.91	0.43	0.57	+3	–3	65.0
5	0.92	0.39	0.61	+3	–3	71.5
6	0.91	0.48	0.52	+3	–3	58.4

**Figure 3:**
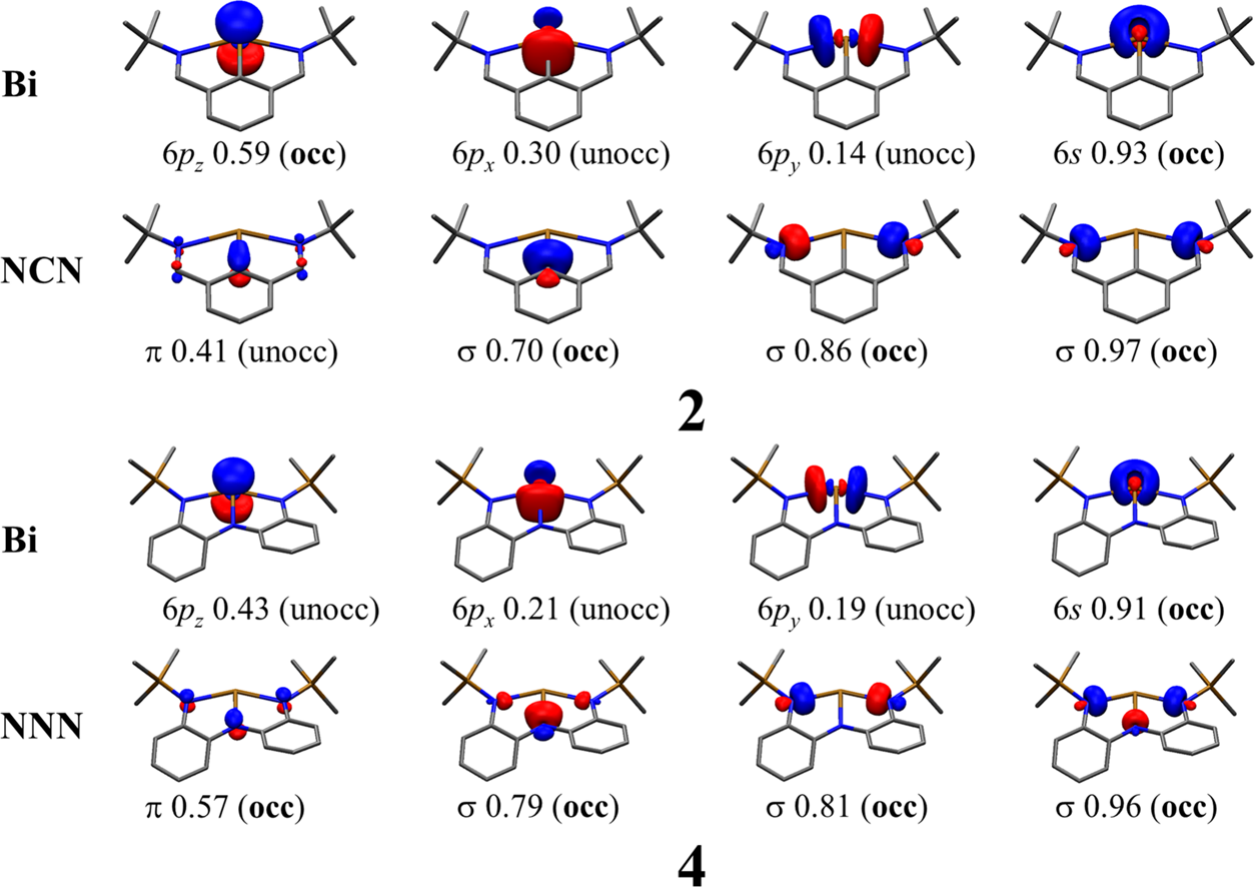
Effective fragment orbitals (EFOs) at the B3LYP-D3(BJ)/def2-TZVPP level of theory for **2** and **4** complexes. The orbital symmetry, gross occupation, and EOS analysis: occupied (occ) and unoccupied (unocc). The isocontour is 0.1 au. Hydrogen atoms are omitted for clarity.

Let us first consider the relatively simple Bi–NCN system **2** from Vránová et al.^23^ EOS analysis gives a picture of Bi(+1) and NCN(−1) with *R* (%) = 68.1. Such values suggest a rather clear OS assignation at the level of theory used. The inspection of the shape and occupation number of the EFOs adds valuable information about the OS assignation process. The most relevant EFOs are depicted in Figure 3. Since the EFOs maintain the σ–π separation, the respective electron distributions separately can be easily visualized. The ligand exhibits three EFOs with σ character toward the Bi center with gross occupations of 0.97, 0.86, and 0.70, respectively. The corresponding orbitals on the Bi atom are formally unoccupied with gross occupations of 0.03 (not shown), 0.14, and 0.30. Thus, with the EFOs’ partitioning, the ligand is considered to have three σ lone pairs, which are coordinating a bismuth atom via dative bonds. The smaller the occupation of the lone pair, the larger the σ-donation from the ligand to the Bi center. The fact that the EFO with a smaller occupation is at the C atom of the ring is in line with the better σ-donating ability of C- than N-ligands.

Concerning the π-bonding, the NCN ligand exhibits five π-type EFOs with an occupation above 0.99, which essentially describe the five π occupied molecular orbitals of the free anionic ligand (see Figure S1 in the Supporting Information). There is an additional π-type EFO that essentially corresponds to the LUMO of the free anionic ligand (Figure 3). It exhibits a gross occupation of 0.41, smaller than that of the p-type orbital on Bi (0.59). Consequently, the EOS analysis considers the latter as formally occupied, which results in a Bi(+1) assignation. Its partial Bi(+3) character originates in π-bonding due to the non-negligible occupation of the ligand’s frontier π-type EFO. Replacing the *t*Bu group of **2** with phenyl derivatives in **1** and **3** has a negligible effect on the EFOs and their occupations, as shown in the Supporting Information (Figures S2 and S3). All of these systems are consistently described as Bi(+1) species.

The consistent NCN(−1) formal charge assignation should not be surprising considering the nature of the ligand. From the isolated ligand perspective, the more plausible formal charge is the one that maintains the aromaticity of the six-membered ring and that corresponds to the (−1) charge. In the hypothetical case, the ligand would gain an electron pair upon fragmentation, the fragment would become formally (−3), but these extra electrons will be located at the π-system, breaking its Hückel aromaticity.^46−48^ The same aromaticity breaking would happen if the ligand would transfer electrons to the metal (see below).

The triamide NNN ligand of compounds **4–6** presents an intriguing situation. There are two plausible anionic states for the NNN ligand, which are associated with the Lewis structures depicted in Figure 1D. In the case of bismuth with an oxidation state of +3, the ligand would carry (−3) of the total charge, with each of the three N-coordinating atoms exhibiting two lone pairs (with σ and π symmetries). In addition, each of the phenyl rings formally bears six π-electrons, as outlined in Figure 1D (**A**). With oxidation state +1, the total charge on the ligand is (−1). This situation is best represented by two resonant Lewis structures, where only one of the coordinating N atoms bears two lone pairs and the remaining N atoms have one lone pair with N → Bi interaction (Figure 1D (**C** and **D**)). The former N atoms are conjugated with the aromatic rings and as a consequence their aromatic character decreases. Nonetheless, there are up to 16 π-electrons that can delocalize among the phenyl rings, which could make the NNN(−1) state plausible.

Notably, EOS analysis for **4** indicates a Bi(+3) center and a formal NNN(−3) ligand with *R* (%) = 65.0, in contrast with a former OS assignment.^26^ The corresponding frontier EFOs are depicted in Figure 3. The shape of the EFOs is very similar to those obtained for the NCN-coordinated system **2**. The σ interaction is split, with the occupations of the ligand-centered EFOs being much higher (0.79, 0.81, 0.96) than those of the 6p-type hybrids on Bi (0.21, 0.19, and 0.04). The higher electronegativity of N (with respect to C) makes the ligand a weaker σ donor, so the 6p_*x*_ occupation of Bi is 0.21 rather than 0.30 as in **2**. The π system shows EFOs analogous to the Bi–NCN system, but here, the occupation of the 6p_*z*_ EFO on Bi (0.43) is smaller than that of the frontier π EFO on the ligand (0.57), which formally keeps the electron pair. There are eight additional π-type EFOs occupied in the ligand, thus leading to the NNN (−3) formal charge and consequently the Bi(+3) assignation (Figure S4).

As mentioned above, Marczenko et al. have explored the substituent effect on the NNN ligand.^26^ We consider here two extreme systems, **5** and **6**, where −CF_3_ and −OCH_3_ substituents, respectively, induce opposite effects on the Lewis acid character of the Bi center. A higher Lewis acid character of Bi should be accompanied by a decrease of its 6p_*z*_ occupation and hence a more marked Bi(+3) character. We have performed EOS analysis on both systems and the occupation of the 6p_*z*_ EFO on Bi decreases from 0.43 for **4** to 0.39 for **5** and increases up to 0.48 for **6**. An opposite trend is observed for the occupation of the ligand’s frontier π EFO.

Note that the assignation of the oxidation state within the EOS approach relies mainly on the dissection of the π-orbital occupation (Figure 3, first column). In most of the cases, the relative occupation of the π frontier EFOs on bismuth and pincer ligand is quite similar. The extreme case is compound **6**, where the occupations are 0.48 for Bi and 0.52 for the NNN fragment, which is translated in a rather small value of the *R* (%) index (54.8). Despite these small differences, the EOS analysis assigns the electron pairs to the ligand, leading into a formal Bi(+3). Note, however, that for the oxidation state assignation of Bi(+1), the occupation dissection is not completely different from the one observed in **1**, where the occupation of the 6p_*z*_-orbital at bismuth is 0.59.

The closed-call OS situation in these systems prompted us to further test the robustness of the assignments. On the one hand, we have studied both basis set and DFT functional dependence of the EOS results for prototypical systems **2** and **4**. The results are gathered in Table S5 of the ESI. We obtain the same OS assignations in all cases, with very small differences in the frontier EFO occupations among the different DFT functionals tested.

On the other hand, we have compared the EOS picture against the one provided by the natural bond orbital method,^49−51^ which has been applied in former studies.^23,26^Table 2 summarizes the contributions of the most relevant localized orbitals involving Bi. More details about the shape and contribution of the NBOs are collected in Tables S8–S17 of the SI.

**Table 2:** NBO Results for **1**–**6** Complexes: C/N–Bi Wiberg Bond Order, NBO Occupations, Orbital Contributions, and Population of Bi’s 6p_*z*_ from the Orbital Contributions of the Bonding and Antibonding π_C/N–Bi_ NBOs

	WBI_C/N–Bi_	Pop. 6p_*z*_ Bi	σ_C/N–Bi_	σ*_C/N–Bi_	π_C/N–Bi_	π*_C/N–Bi_
1	1.09	1.43	1.95, Bi(30%)–C(70%)	0.05, Bi(70%)–C(30%)	1.83, Bi(67%)–C(33%)	0.62, Bi(33%)–C(67%)
2	1.11	1.40	1.95, Bi(30%)–C(70%)	0.05, Bi(70%)–C(30%)	1.82, Bi(65%)–C(35%)	0.62, Bi(35%)–C(65%)
3	1.12	1.40	1.95, Bi(31%)–C(69%)	0.05, Bi(69%)–C(31%)	1.82, Bi(65%)–C(35%)	0.61, Bi(35%)–C(65%)
4^a^	0.68	1.00	1.95, Bi(16%)–N(84%)	0.13, Bi(84%)–N(16%)	1.78, Bi(27%)–N(73%)	0.71, Bi(73%)–N(27%)
5^a^	0.70	0.89	1.95, Bi(16%)–N(84%)	0.11, Bi(84%)–N(16%)	1.78, Bi(24%)–N(76%)	0.62, Bi(76%)–N(24%)
6^a^	0.67	1.12	1.95, Bi(16%)–N(84%)	0.16, Bi(84%)–N(16%)	1.80, Bi(32%)–N(68%)	0.80, Bi(68%)–N(32%)

a Enforced Lewis structure with lower non-Lewis density % value.

The orbital localization leads in all cases to a 6s-type bismuth lone pair with an occupation of ca. 2 electrons, as described by Vránová et al.^23^ In systems **1**–**3**, the σ-type interaction between Bi and the pincer ligand is represented by one lone pair on each N atom and a two-electron Bi–C bond polarized toward the ligand’s C atom. In addition, we obtain a bonding Bi–C π-bond polarized toward Bi with an occupation of ca. 1.8, and the corresponding antibonding NBO with the reversed bond polarization and an occupation of ca. 0.60. This clear Bi(+1) picture is in perfect agreement with our EOS results.

It is worth pointing out that our results for **3** differ from those obtained by Vránová et al. for the same system,^23^ where instead of a Bi–C π-bond they obtain a fully localized 6p_*z*_ orbital on Bi with an occupation of 1.35. By enforcing in the NBO analysis^52,53^ to include a Bi 6p_*z*_ lone pair into the Lewis structure, we essentially recovered Vránová results (see Table S14), leading to a non-Lewis density value (2.35%) somewhat larger than that of the default calculation (2.19%). Both pictures reconcile by quantifying the population of the Bi 6p_*z*_ natural atomic orbital from the bonding and antibonding Bi–C π-bonds, as gathered in Table 2. Nonetheless, in our opinion, the two-electron bonding/antibonding NBO description permits a much closer connection with IUPAC’s winner-takes-all principle (in line with the LOBA^32^ approach for OS assignation).

The 6p_*z*_ lone-pair picture also emerged by default for system **4** with an occupation as low as 1.0 (Table S11), in perfect agreement with the results reported by Kindervater et al.^26^ However, the default NBO analysis of complexes **5**–**6** lead instead to a pair of bonding and antibonding Bi–N π-bonds clearly polarized toward the ligand’s atom and to some minor differences in the σ-bonding involving Bi (lone-pair vs strongly polarized bond, see Tables S12 and S13).

According to Marczenko et al.^27^ and to EOS analysis, one would expect the Bi(+3) character of **4** to lie somewhat in between **5** and **6**. This is precisely what could be inferred from the population of the calculated Bi 6p_*z*_ orbital in Table 2. Moreover, the WBI_N-Bi_ values for **4**–**6** are very similar (the same as among **1**–**3**), which does not seem to indicate that a significantly different picture is expected for **4**, **5**, and **6**. We then opted for an enforced NBO analysis for **4–6** leading to a picture analogous to that obtained for **1**–**3**, that is, including a pair of bonding and antibonding Bi–N π-bonds and the two lone pairs on the N centers. To our surprise, the non-Lewis density values were smaller than those obtained by the default calculations in all cases (see Tables S15–S17).

So, it appears that different formal pictures (not necessarily associated with the lowest non-Lewis density value) can be obtained with NBO analysis by default, which hinders the comparison of the bonding situation among Bi–NCN and Bi–NNN systems. Considering the same NBO solution for all systems (which is also the one with lower non-Lewis density values) clearly confirms that the Bi–C π-bond polarity in **1**–**3** (toward Bi) is completely reversed in the case of **4**–**6** (toward N), in full agreement with EOS. A clear advantage of EOS analysis for these systems is that it readily permits a straight comparison of the electronic structure of all systems on equal footing, independently of the dominant Lewis structure.

To further corroborate the relationship between the occupation of the EFOs and the Lewis base properties, we have computed the first and the second proton affinities for compounds **1**–**6**. Previous studies have shown that the first and second proton affinities (PAs) are sensitive probes for the presence of chemically available lone pairs of a molecule.^55−59^ Thus, the values provide information about the location and the ability of the lone pairs to coordinate Lewis acids.

Table 3 gathers the calculated PAs of **1**–**6** at the B3LYP-D3(BJ)/def2-TZVPP level of theory. The first PAs of all compounds, but **5**, are higher than 220 kcal/mol, which suggests a highly basic nature. Note that the calculated PA values follow the trend of the occupation of the 6p_*z*_ EFOs of the Bi atom. The highest PA is for **3** (249.6 kcal/mol) with a 6p_*z*_ occupation of 0.60. At the other extreme, compounds **5** has a PA (188 kcal/mol) and an occupation of 0.39. The first PA also closely follows the trends of the 6s and 6p_*z*_ natural atomic orbital (NAO) energies, in line with the findings of Chval et al.^60^ for donor–acceptor adducts driven by electrostatic interactions.

**Table 3:** First and Second Proton Affinities (PAs)^a^ and Bond Dissociation Energies Including ZPE Corrections of 1–6 with One and Two W(CO)_5_ and HNMe_2_ (*D*_0_)^b^^,^^c^^,^^d^^,^^e^

	*n* = 1							*n* = 2						
system	PA/*D*_0_	6s Bi	6p_*z*_ Bi	π L	OS Bi	OS L	*R* (%)	PA/*D*_0_	6s Bi	6p_*z*_ Bi	π L	OS Bi	OS L	*R* (%)
1-(H^+^)*_n_*	244.2	0.92	0.34	<0.05	+3	–1	75.8	103.1	0.69	0.33	<0.05	+3	–1	50.0
1-(W(CO)_5_)*_n_*	48.1	0.92	0.53	0.20	+1	–1	71.0	33.2	0.87	0.55	0.13	+1	–1	78.3
2-(H^+^)*_n_*	243.3	0.92	0.35	<0.05	+3	–1	75.4	94.4	0.69	0.33	<0.05	+3	–1	50.0
2-(W(CO)_5_)*_n_*	52.0	0.92	0.54	0.19	+1	–1	73.4	37.1	0.88	0.53	0.10	+1	–1	77.7
3-(H^+^)*_n_*	249.6	0.92	0.36	<0.05	+3	–1	75.5	114.5	0.70	0.34	<0.05	+3	+1	53.6
3-(W(CO)_5_)*_n_*	53.4	0.92	0.55	0.18	+1	–1	74.1	44.1	0.87	0.52	0.10	+1	–1	76.5
4-(H^+^)*_n_*	220.4	0.90	0.34	<0.05	+3	–1	75.9	98.4	0.68	0.33	<0.05	+3	–1	71.6
4-(W(CO)_5_)*_n_*	38.7	0.90	0.48	0.31	+1	–1	65.6	34.5	0.88	0.51	0.18	+1	–1	80.2
4-(HNMe_2_)*_n_*	12.1	0.90	0.28	0.74	+3	–3	97.4	16.4	0.88	0.18	0.87	+3	–3	100
5-(H^+^)*_n_*	188.0	0.91	0.36	<0.05	+3	–1	73.7	62.5	0.69	0.34	<0.05	+3	–1	67.6
5-(W(CO)_5_)*_n_*	27.8	0.91	0.46	0.38	+1	–1	56.1	30.7	0.88	0.51	0.20	+1	–1	77.0
5-(HNMe_2_)*_n_*	20.4	0.91	0.23	0.85	+3	–3	100	21.2	0.88	0.18	0.91	+3	–3	100
6-(H^+^)*_n_*	229.1	0.90	0.35	<0.05	+3	–1	76.4	100.5	0.69	0.33	<0.05	+3	–1	72.8
6-(W(CO)_5_)*_n_*	41.9	0.90	0.51	0.25	+1	–1	73.0	39.7	0.86	0.52	0.13	+1	–1	76.7
6-(HNMe_2_)*_n_*	8.0	0.90	0.36	0.63	+3	–3	77.3	11.3	0.88	0.21	0.83	+3	–3	100

a The proton affinities are defined as PA_1_ = Δ*H*(**1**–**6**) + Δ*H*(H^+^) – Δ*H*(**1**–**6-**(H^+^)) and PA_2_ = Δ*H*(**1**–**6-**(H^+^)) + Δ*H*(H^+^) – Δ*H*(**1**–**6-**(H^+^)_2_) as described in ref (54). Proton enthalpy +1.5 kcal/mol.

b Frontier EFOs occupations (in au) of Bi and the pincer ligand (NCN or NNN) and assigned oxidation states.

c All calculations were performed at the B3LYP-D3(BJ)/def2-TZVPP level of theory.

d All energies are in kcal/mol.

e Three pseudodegenerated EFOs (in occupation), one from the NCN pincer ligand and two from H atoms (one each).

Applying EOS analysis on compounds **1–6-(H**^**+**^**)** shows a clear picture with Bi(+3), NCN/NNN(−1), and H(−1) assignation. Such situation results from the different electronegativity of H and Bi, which implies formal oxidation of the Bi center to Bi(+3), while the H moiety is pictured as a hydride (−1). Bi(+3)-hydride **2-(H**^**+**^**)** was postulated as an intermediate in the catalytic dehydrogenation of ammonia-borane with **2**. This species was detected by high-resolution mass spectrometry (MS), but all attempts for its isolation were unsuccessful.^5^ Noteworthily, regardless of the formal nature of the Bi center (+1 in **1**–**3** and +3 in **4**–**6**), we observe in all cases a full decay of the π-EFO occupation (<0.05) of the ligand upon hydride formation. In **1**–**3**, Bi is electronically rich enough and readily provides the electron pair to form the hydride, thereby formally oxidizing to +3. In **4**–**6**, it is mainly the NNN ligand that provides the electrons to form the hydride.

Table 3 also gathers the calculated values for the second PA of the molecules, which are particularly important for testing the coordinating ability of the second lone pair and hence the Bi(+1) character. The values are relatively high (ca. 100 kcal/mol) and comparable to those reported for divalent ylidone E(0) compounds.^55−59^ The reported values for the elusive Pb(0) species are 273.8 and 114.9 kcal/mol for the first and second proton affinities, respectively.^55^ The correlation with the Bi’s 6p_*z*_ occupation of the deprotonated species is not as good as for the first PA. In fact, the second PA should probe the second available lone pair on Bi, which corresponds to a 6s-type EFO exhibiting a large and constant occupation of ca. 0.90 for all species. This explains why the second PA is rather constant among the systems studied, no matter their formal OS is Bi(+1) or Bi(+3). Species **5** is the only exception, for which both the first and second PAs are somewhat smaller than for the rest of the systems, in line with its weakest Lewis basic character. Noteworthily, the energies of the 6s and 6p_*z*_ NAO of the monoprotonated species do follow closely the trend of the second PA.

Our calculations suggest that both formal Bi(+1) and Bi(+3) are able to coordinate two strongly polarizing Lewis acids. The second protonation is likely to be experimentally unachievable, considering that already the single Bi(+3)-hydride has not yet been isolated. EOS analysis of these species also indicate only partial hydride character of the H moieties, as Bi remains with the formal OS of (+3) in all cases.

We also considered the adducts with the electron-deficient W(CO)_5_ species (**1–6-(W(CO)**_**5**_).^23^ The successful synthesis of tungsten complexes is typically used as an experimental signature of Bi(+1) character, where the available 6p_*z*_ electrons of Bi are used to form a dative Bi → W bond. Hence, upon reaction with the W(CO)_5_ Lewis acid, the Bi center should formally remain Bi(+1). Indeed, the results of the EOS analysis are in full agreement with these considerations. For instance, for **2-(W(CO)**_**5**_**)** EOS gives a clear Bi(+1), NCN(−1) and W(CO)_5_(0) assignment, with *R* (%) = 73.4. The OS assignation is driven by newly formed bond, as the σ interaction between Bi and NCN ligand remains essentially unchanged (see most relevant EFOs in Figure S5). However, when bonded to the W(CO)_5_ unit, the occupation of the 6p_*z*_ EFO on Bi slightly decreases from 0.59 (**2**) to 0.54e (**2-W(CO)**_**5**_). Also, the occupation of the frontier π EFO on the ligand drops from 0.41 (**2**) to 0.19 (**2-W(CO)**_**5**_). These electrons are used to populate the otherwise empty σ-type EFO on the W(CO)_5_ moiety (0.29). Still, the large occupation of the 6p_*z*_ EFO of Bi indicates its predominant Bi(+1) character. The π-density of the NCN ligand is significantly altered, but still the ligands act formally as a spectator in both species.

The aforementioned OS assignation of species **4**–**6** implies that the 6p_*z*_ lone pair on Bi is formally absent, so they could potentially exhibit different reactivity toward Lewis acid and protonation than **1**–**3**. However, adduct **4-W(CO)**_**5**_ was observed and characterized by MS and NMR spectroscopy,^28^ which could be in an apparent contradiction to the Bi(+3) assignment.

Notably, the dissociation energies of the adducts **1**–**6** exhibit again an excellent correlation with the Bi 6p_*z*_ occupation of the precursor, no matter the formal OS of the Bi center. Thus, the smaller the occupation, the smaller the *D*_0_ value, in line with a more pronounced Bi(+3) character.

**Figure 4:**
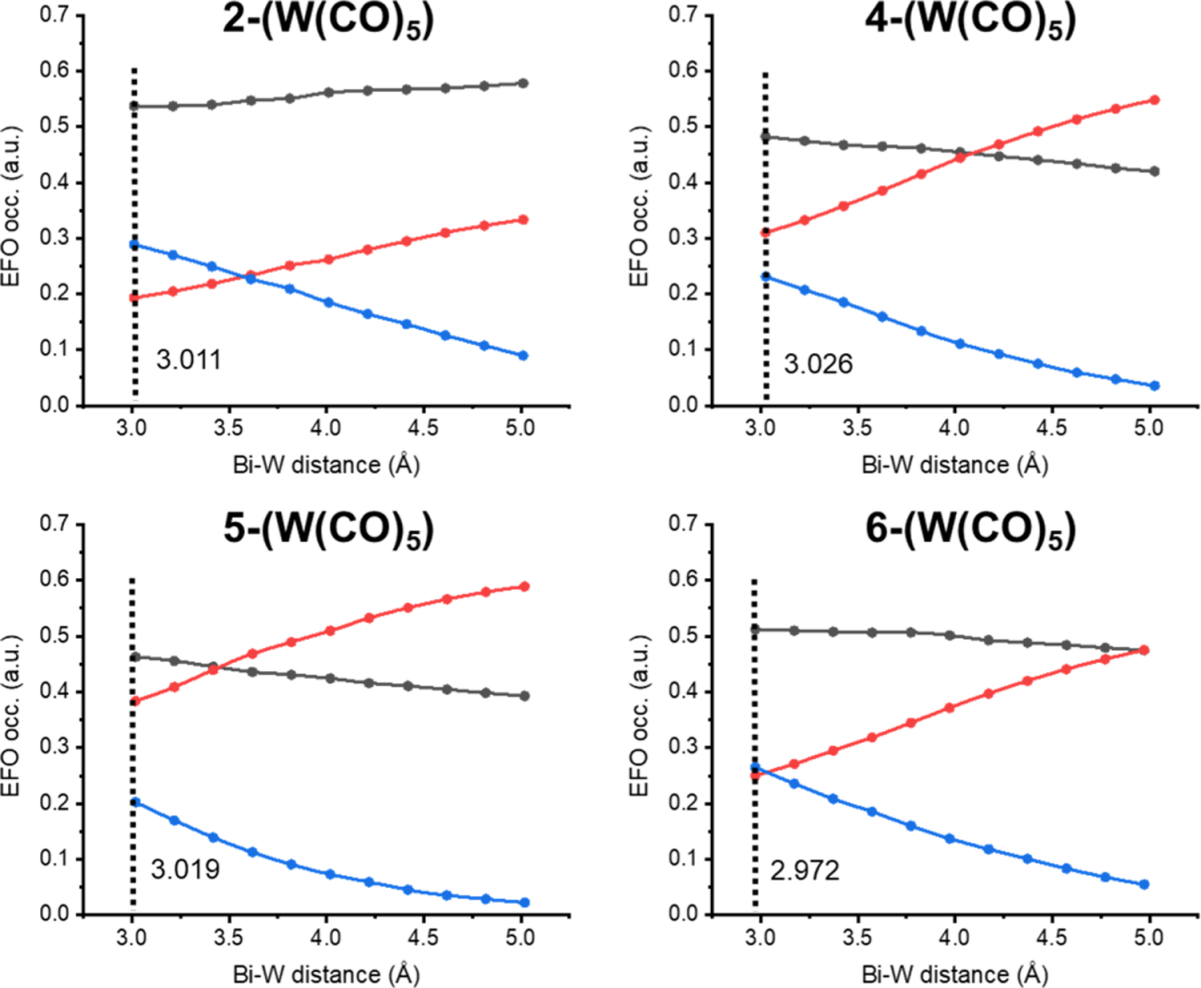
Gross EFO occupations along the Bi–W bond distance for species **2-(W(CO)**_**5**_**)**, **4-(W(CO)**_**5**_**)**, **5-(W(CO)**_**5**_**)**, and **6-(W(CO)**_**5**_**)**. Bi 6p_*z*_ (grey line), NCN/NNN π-type (red line) and W σ-type (blue line).

For further illustration, Figure 4 depicts the gross occupation evolution of the relevant EFOs along the Bi–NCN/N···W(CO)_5_ dissociation profile. For **2-(W(CO)**_**5**_**)**, when the metal approaches Bi, the σ-EFO of the W(CO)_5_ moiety pointing toward Bi steadily increases its occupation from essentially zero (5.0 Å) to ca. 0.2 in equilibrium (3.011 Å). This accounts for the modest donation from Bi to W. The small value is consistent with a dative picture of the Bi–W bond. However, the occupation of Bi’s 6p_*z*_ EFO remains rather constant along the profile (gray curve) and hence the Bi(+1) character is kept. On the contrary, it is the occupation of the ligand’s π EFO (red curve in Figure 4) that steadily decreases as the Lewis acid W(CO)_5_ approaches.

The same mechanism occurs in **4–6-(W(CO)**_**5**_**)** adducts. However, since in **4**–**6** the NNN ligand has a formal (−3) charge, the adduct formation implies formal oxidation of the ligand and reduction to Bi(+1). Figure 4 shows the π EFO occupation of NNN (red curve) steadily decreasing from its large value for the isolated Bi–NNN species (5.0 Å) to a value below that of Bi’s 6p_*z*_ (gray curve) upon adduct formation. The crossing point corresponds to the formal change of OS from Bi(+3) to Bi(+1) and the corresponding oxidation of NNN. The occupation of the 6p_*z*_ EFO of Bi slightly increases upon adduct formation, but the electron pair of the new Bi → W bond essentially comes from the ligand’s π system, which again explains the fact that these adducts are stable regardless of the formal OS of the Bi center of the precursor. The location of the crossing point in Figure 4 is in line with Lewis basic character of the latter. Thus, the formal change of OS upon coordination occurs close to equilibrium distance for the least Lewis basic species **5** (3.3 Å), followed by **4** (4.0 Å) and **6** (5.0 Å). Note that coordination to a second W(CO)_5_ is thermodynamically plausible, despite no experimental evidence has been reported. The data in Table 3 clearly indicates that with the second W(CO)_5_ unit the occupation of the π EFO of the ligand further halves, while that of Bi’s 6p_*z*_ EFO remains essentially constant.

We have also considered the coordination of species **4**–**6** with one and two units of dimethylamine (HNMe_2_). The low *D*_0_ values obtained suggest a rather labile Lewis pair. The release HNMe_2_ has been experimentally observed by Kindervater et al. for the preparation of **4** from **4-(HNMe**_**2**_**)**_**2**_.^26^ The authors argued that the deamination leads to a reduction of the original Bi(+3) center to Bi(+1) by concomitant oxidation of the pincer ligand that would provide the electron pair, but according to our calculation, no change on the oxidation state is observed. EOS analysis of the mono- and diaminated species points to an undisputed Bi(+3) NNN(−3) character, especially for the diaminated ones. The occupation of the π EFO of the NNN ligand steadily increases going from **4** (0.57) to **4-(HNMe**_**2**_**)** (0.74) and to **4-(HNMe**_**2**_**)**_**2**_ (0.87), indicating that it is the π system of the ligand that collects the excess electrons coming from the σ-donating amines. Such substantial change in occupation is concomitant with a structural deformation of NNN that points toward a certain dearomatization of the phenyl rings upon deamination, as noted by Kindervater et al.^26^ Comparing the results for species **4**–**6**, we observe a decrease of the *D*_0_ values with the occupation of the 6p_*z*_ EFO on Bi, supporting the relationship between the EFO occupations with the Lewis acid/base character.

**Figure 5:**
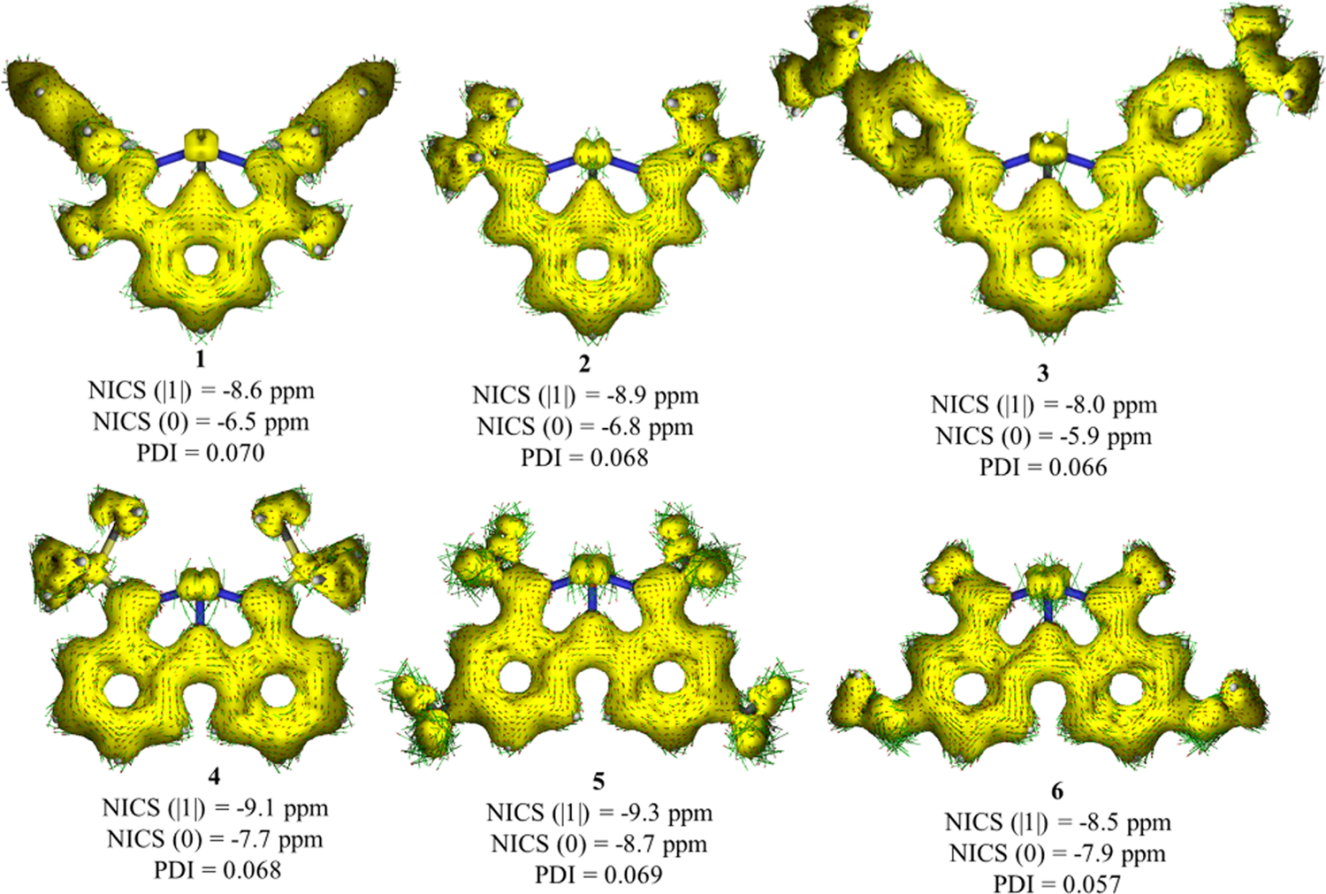
AICD plot of **1**–**6** at B3LYP-D3(BJ)/def2-TZVPP isosurface values (0.03 au) together with NICS (in ppm) and PDI values of the phenyl rings. For AICD, clockwise and counterclockwise circulations suggest diatropicity and paratropicity, respectively. Computed reference values (benzene): NICS (|1|) = −10.1 ppm, NICS (0) = −8.2 ppm, PDI = 0.098.

Finally, Vránová et al.^25^ studied the aromaticity of **2** by means of the magnetic indicator nucleus-independent chemical shift (NICS(−1), NICS(0), and NICS(+1)), finding that the phenyl ring was clearly aromatic. To assess the π-conjugation and magnetic properties of **1–6**, we performed anisotropy of induced current density (AICD),^61^ NICS,^62^ and the electronic para-delocalization index (PDI)^63,64^ analyses (see the Computational Details section). Figure 5 shows the results on the aromaticity indexes for compounds **1–6**. We find NICS rather inconvenient for these systems that involve rather bulky ligands that can alter their numerical values, especially for the nonplanar systems. Moreover, except for the very symmetric species, the value of NICS (1, −1) depends upon the direction from the geometric center of the ring (see Supporting Information Table S6). Therefore, we report the average of the two options as NICS (|1|). On the contrary, a much simpler electronic descriptor such as PDI can better capture the subtle changes in aromaticity.

Comparing the aromaticity indices obtained with reference values for benzene, one can clearly identify the analyzed rings as aromatic. The PDI values for species **1–3** are very similar, in line with the almost constant occupation of ca. 0.40 of the π-type EFO of the respective ligand. More significant changes are observed upon adduct formation or protonation. For instance, the PDI values for **2-(W(CO)**_**5**_**)** and **2-(H**^**+**^**)** species increase up to 0.077 and 0.083, respectively (see SI Table S6). At the same time, the occupation of the π-type EFO **in 2-(W(CO)**_**5**_**)** and **2-(H+)** decreases to 0.19 and <0.05, respectively. Thus, the smaller the occupation of the ligand frontier π EFO, the more the NCN(−1) character and, consequently, the larger the aromaticity of the ring.

A similar trend is observed for species **4**–**6**. In this case, however, the larger the occupation of the ligand frontier π EFO, the more the NNN(−3) character and the larger the aromaticity. The PDI value of the rings in **6** is as low as 0.057, in line with the smaller π EFO occupation (0.52) and its larger share of partial NNN(−1) character. Also, protonation and adduct formation induce a decrease of the ligand’s π EFO occupation (and a formal reduction of the ligand), which contrary to **1**–**3** leads to a decrease of the aromaticity.

## Conclusions

The intriguing Bi(+1)/Bi(+3) redox couple on pincer complexes represents a challenging example for traditional oxidation state assignation based on the reactivity pattern. We have shown that the effective fragment orbitals and the effective oxidation states analysis affords a scrutiny of the electronic structure of the complexes from ground-state properties, i.e., without recurring to reference states. The application of this method on bismuthinidene bis(ketimine)phenyl (NCN) and triamide bismuthinidene (NNN) pincer complexes results in a different oxidation state for the central bismuth atom, being Bi(+1) and Bi(+3), respectively. However, regardless of the formal oxidation state, all complexes are able to react with a series of Lewis bases and acids. The ambiphilic behavior of these complexes is a direct consequence of the strong π-conjugation between the bismuth atom and the pincer ligand. Interestingly, such reactivity can be quantitatively assessed by Bi’s 6p_*z*_ effective fragment orbital occupation.

## Computational Details

All geometry optimizations were performed using the B3LYP density functional^65,66^ in combination with the def2-TZVPP basis set for H, C, N, O, F, and Si atoms.^67^ For bismuth, a def2-TZVPP basis was combined with the def-ECP pseudopotential.^68^ Normal mode analyses were computed to confirm minima on the potential energy surface and to calculate unscaled zero-point energies (ZPEs) as well as thermal corrections and entropy effects using the standard statistical–mechanical relationships for an ideal gas.^69^ All DFT calculations were performed with the Gaussian16 package,^70^ including in all cases the empirical dispersion correction of Grimme (D3),^71^ together with the Becke–Johnson (BJ) damping function.^72^

Spin-resolved effective fragment orbitals (EFOs) and subsequent EOS analyses have been performed with the APOST-3D program.^73^ The topological fuzzy Voronoi cells (TFVC)^74^ atomic definition, a fuzzy-atom efficient and robust real-space alternative to QTAIM, has been used. The sum of the occupations of the EFOs of each fragment equals the fragment’s *net* population. Gross occupations adding up to the total fragment population^36,37^ have been used throughout.

Aromaticity has been evaluated by means of the (magnetic) nuclear-independent chemical shift (NICS)^63^ and the (electronic) para-delocalization index (PDI).^64^ NICS values correspond to the negative value of the absolute shielding computed at the geometric ring center (NICS(0)) or at a distance above and below it and perpendicular to the ring plane. An extensively used distance value is 1 Å above (NICS(1)) and below (NICS(−1)). The larger (and negative) the value, the more aromatic the ring. The PDI is defined as the average of the bond order between atoms in the para position of the ring. Thus, it can only be applied to evaluate the aromaticity of six-membered rings. Large and positive values are obtained for aromatic rings. The NICS results have been obtained using the gauge-including atomic orbital method (GIAO)^75,76^ from Gaussian16, while the PDI values were obtained with APOST-3D.

## Supporting Information Available

Graphical representation of the relevant EFOs and their occupation for all species (Figures S1–S9), numerical data associated with Figure 4 (Tables S1–S4), benchmark of EOS analysis for **2** and **4** with several KS-DFT functionals (Table S5), aromaticity indicators for species **1**–**6** (Table S6), EOS analysis benchmark of Bi-based compounds (Table S7), and NBO analysis for species **1**–**6** (Tables S8–S17) (PDF)
Cartesian coordinates of all optimized species (XYZ)

## Supplementary Material

ic1c02252_si_001.pdf

ic1c02252_si_002.xyz
