# Supplementary material for: Unveiling the Electronic Structure of the Bi(+1)/Bi(+3) Redox Couple on NCN and NNN Pincer Complexes
Source: Inorg Chem. 2021 Nov 12;60(23):17657–68. doi: 10.1021/acs.inorgchem.1c02252 (PMC8653152; doi:10.1021/acs.inorgchem.1c02252)
Supplement: Supplementary file 1 — ic1c02252_si_001.pdf [file ic1c02252_si_001.pdf]

Supporting Information

for

Unveiling the Electronic Structure of the Bi(+1)/Bi(+3) Redox Couple on NCN and NNN Pincer Complexes

Martí Gimferrer,[1] Sergi Danés,[1,2] Diego M. Andrada[2,*] and Pedro Salvador[1,*]

1) Institut de Química Computacional i Catàlisi and Departament de Química, Universitat de Girona, Maria Aurèlia Capmany 69, 17003 Girona, Catalonia, Spain.

2) Saarland University, Faculty of Natural Sciences and Technology, Department of Chemistry, 66123 Saarbrücken, Federal Republic of Germany.

Email: diego.andrada@uni-saarland.de, pedro.salvador@udg.edu

The supporting information contains:

Figure S1. π-type NCN-ligand EFOs shape and information for system 2 …………………………………..…………. S2
Figure S2. Valence EFOs shape and information for system 1 ………………………………………………………..… S3
Figure S3. Valence EFOs shape and information for system 3 ………..……..………………………….……….…. S4
Figure S4. π-type NNN-ligand EFOs shape and information for system 4 ……………………………..……..…S5
Figure S5. Valence EFOs shape and information for system 2-($W(CO)_5$) …………………….…………..………….... S6
Figure S6. Valence EFOs shape and information for system 2-(H^+) ……………..…………………………….……..… S7
Figure S7. Valence EFOs shape and information for system 4-($(HNMe_2)_2$) …………………………………..……..…S8
Figure S8. Valence EFOs shape and information for system 5 ……………………………………………………..…S9
Figure S9. Valence EFOs shape and information for system 6 …………………………………………………….…S10
Table S1. Bi-W distance, electronic energy, fragment gross populations and oxidation states along the Bi-W bond breaking potential energy surface for system 2-($W(CO)_5$) ………………………………………………………………….. S11
Table S2. Bi-W distance, electronic energy, fragment gross populations and oxidation states along the Bi-W bond breaking potential energy surface for system 4-($W(CO)_5$) ………………………………………………………………….. S11
Table S3. Bi-W distance, electronic energy, fragment gross populations and oxidation states along the Bi-W bond breaking potential energy surface for system 5-($W(CO)_5$) ………………………………………………………………….. S12
Table S4. Bi-W distance, electronic energy, fragment gross populations and oxidation states along the Bi-W bond breaking potential energy surface for system 6-($W(CO)_5$) ………………………………………………………………….. S12
Table S5. Benchmark of KS-DFT functionals and EOS analysis for systems 2 and 4…………………………………. S13
Table S6. Aromaticity indications (NICS and PDI) for systems 1-6, 1-(H^+)-6-(H^+) and 1-($W(CO)_5$)-6-($W(CO)_5$)…… S13
Table S7. Benchmark of Bi-based molecular systems and its corresponding EOS analysis evaluated at the B3LYP(D3-BJ)/def2-TZVPP level of theory………………………………………………………………………………S14-S16
Tables S8-S17. NBO results for systems 1-6……………………………………………………………………… S17-S35

References ……..…………………………………………………………………………………………………...S36

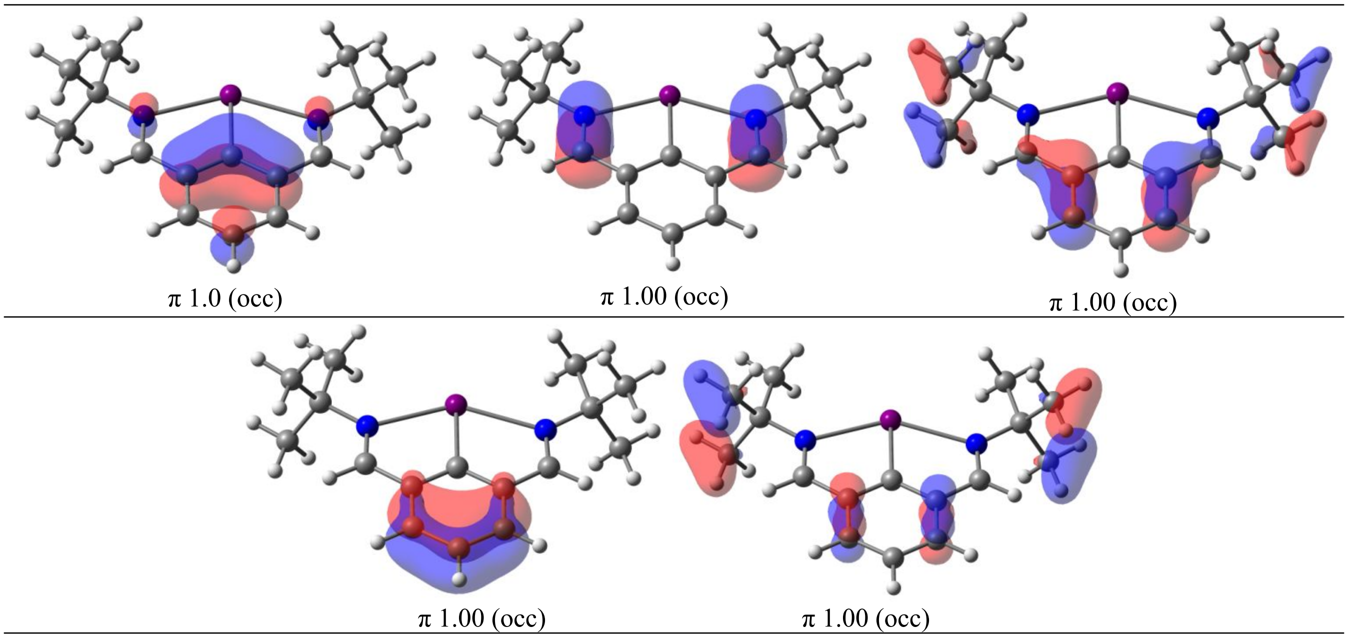

Figure S1. π-occupied NCN-ligand Effective Fragment Orbitals (EFOs) at the B3LYP-D3(BJ)/def2-TZVPP level of theory for 2 with gross populations larger than 0.95. Isocontour value of 0.05 a.u.

**Bi**	**NCN-ligand**
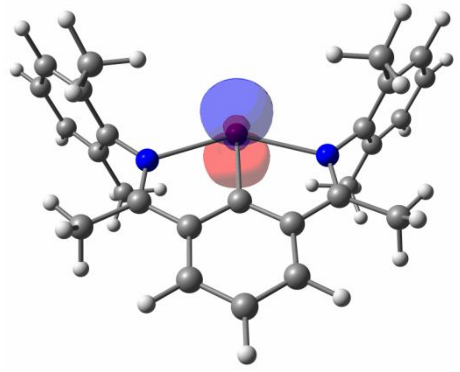 6p_z 0.60 (occ)	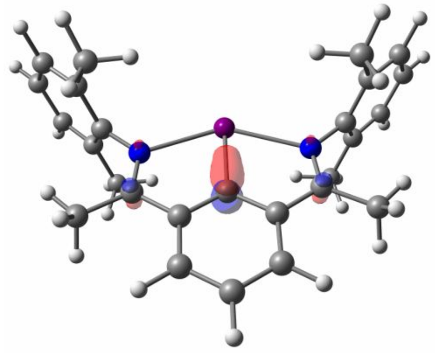 π 0.41 (unocc)
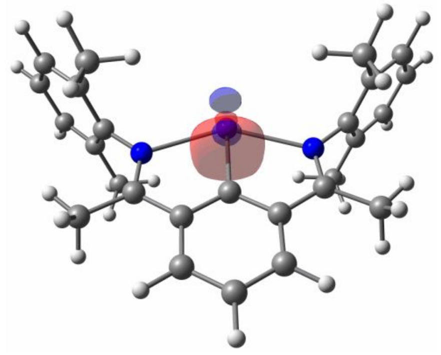 6p_x 0.30 (unocc)	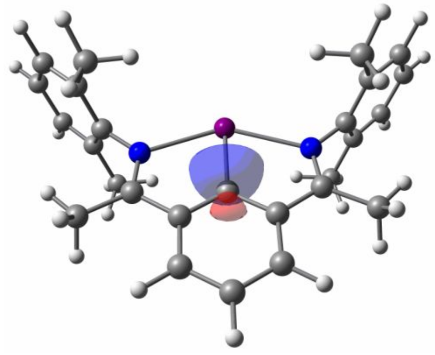 σ 0.70 (occ)
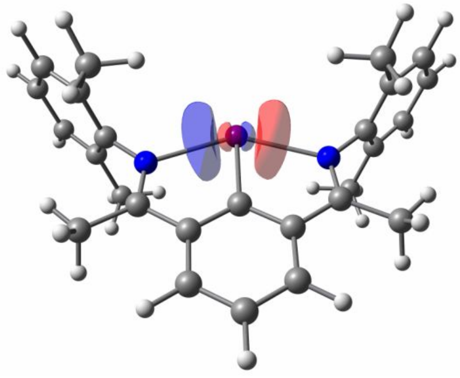 6p_y 0.14 (unocc)	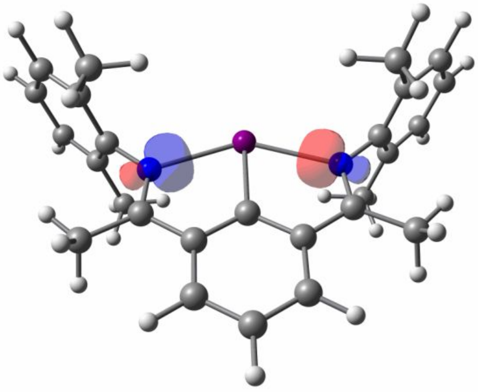 σ 0.86 (occ)
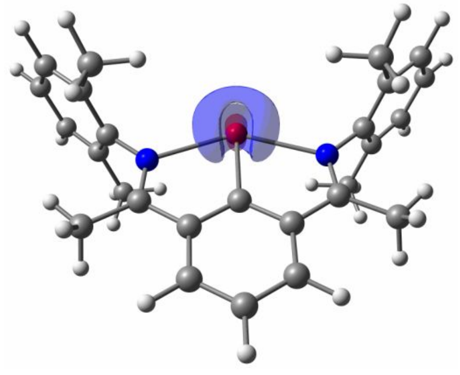 6*s* 0.93 (occ)	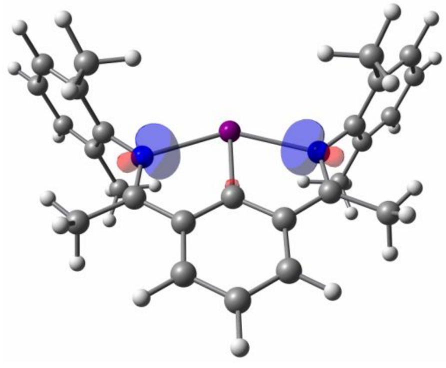 σ 0.97 (occ)

Figure S2. Valence Effective Fragment Orbitals (EFOs) at the B3LYP-D3(BJ)/def2-TZVPP level of theory of the Bi (left) and NCN-ligand (right) fragments for 1, together with symmetry, gross population and result of EOS analysis (occ/unocc). Isocontour value of 0.1 a.u.

**Bi**	**NCN-ligand**
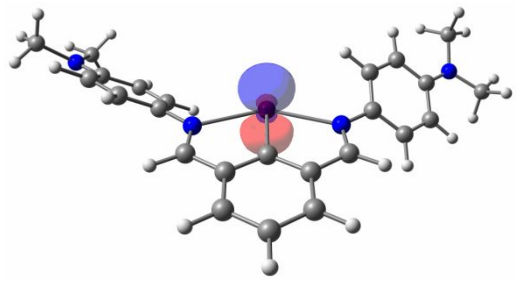 6p_z 0.60 (occ)	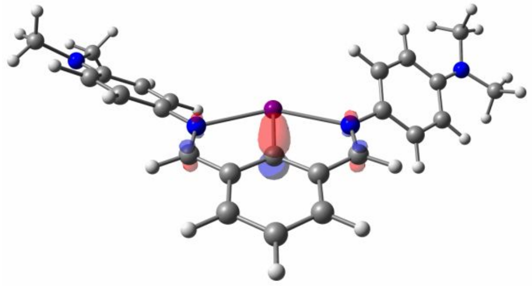 π 0.40 (unocc)
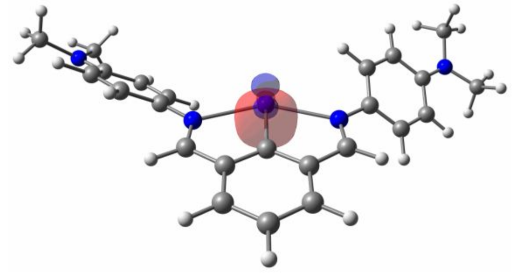 6p_x 0.30 (unocc)	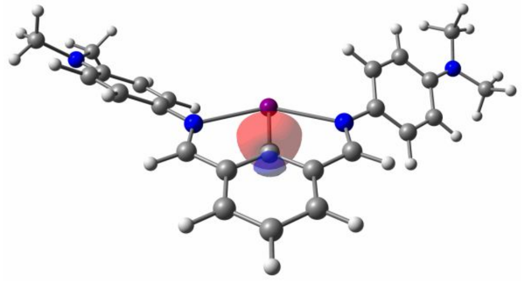 σ 0.70 (occ)
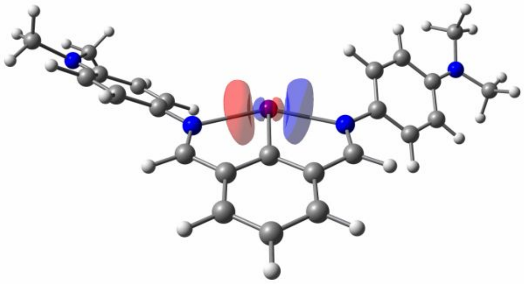 6p_y 0.14 (unocc)	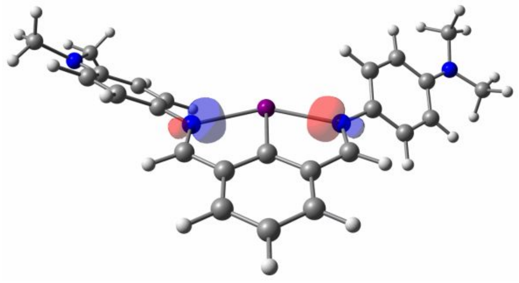 σ 0.86 (occ)
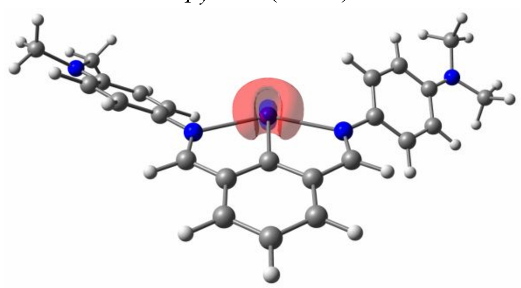 6*s* 0.93 (occ)	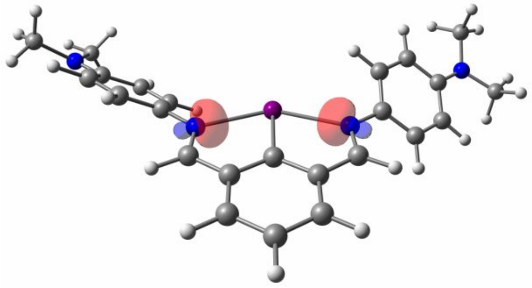 σ 0.97 (occ)

Figure S3. Valence Effective Fragment Orbitals (EFOs) at the B3LYP-D3(BJ)/def2-TZVPP level of theory of the Bi (left) and NCN-ligand (right) fragments for 3, together with symmetry, gross population and result of EOS analysis (occ/unocc). Isocontour value of 0.1 a.u.

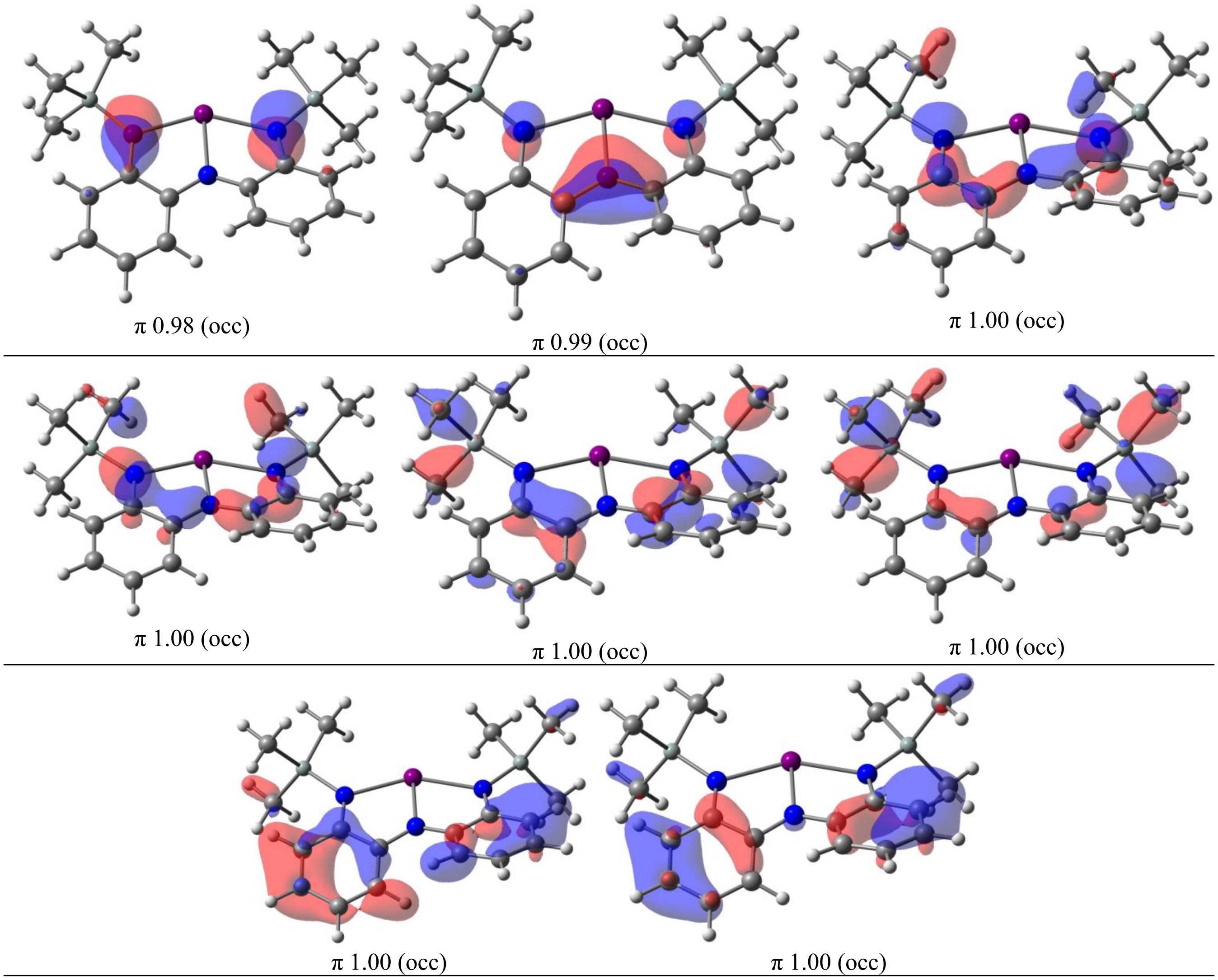

Figure S4. π-occupied NNN-ligand Effective Fragment Orbitals (EFOs) at the B3LYP-D3(BJ)/def2-TZVPP level of theory for 4 with gross populations larger than 0.95. Isocontour value of 0.05 a.u.

**Bi**	**NCN-ligand**	**$W(CO)_5$**
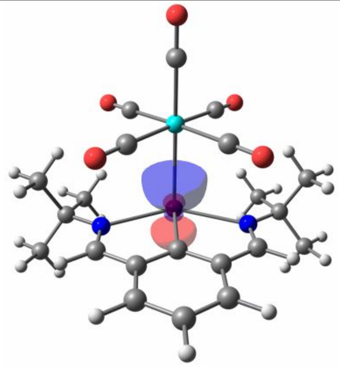<br>$6p_z$ 0.54 (occ)	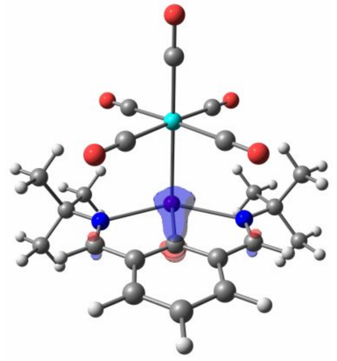<br>π 0.19 (unocc)	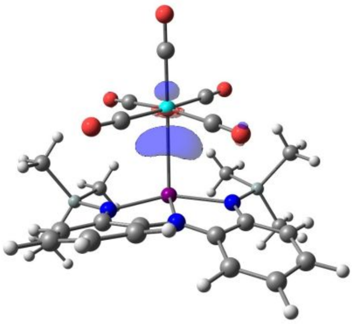<br>σ 0.29 (unocc)
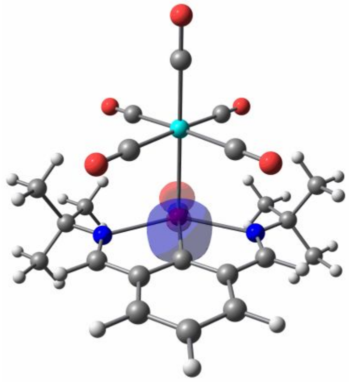<br>$6p_x$ 0.29 (unocc)	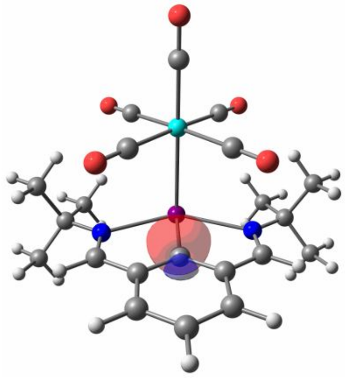<br>σ 0.71 (occ)	
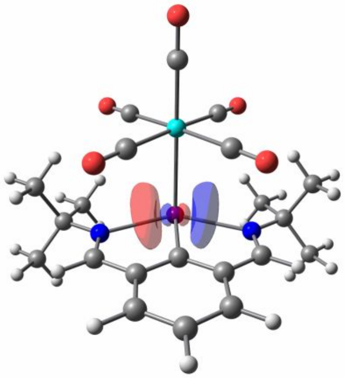<br>$6p_x$ 0.15 (unocc)	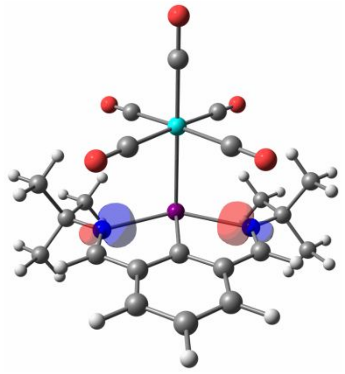<br>σ 0.86 (occ)	
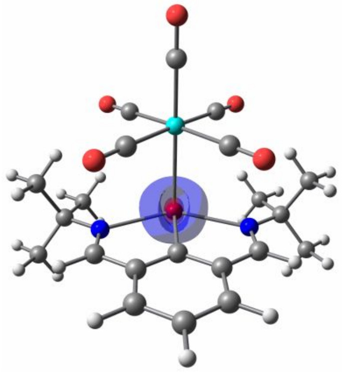<br>$6s$ 0.92 (occ)	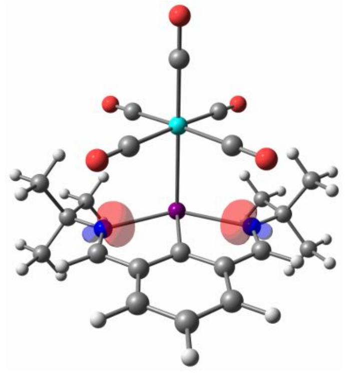<br>σ 0.97 (occ)	

Figure S5. Valence Effective Fragment Orbitals (EFOs) at the B3LYP-D3(BJ)/def2-TZVPP level of theory of the Bi (left) and NCN-ligand (middle) and $W(CO)_5$ (right) of 2-($W(CO)_5$), together with symmetry, gross population and result of EOS analysis (occ/unocc). Isocontour value of 0.1 a.u.

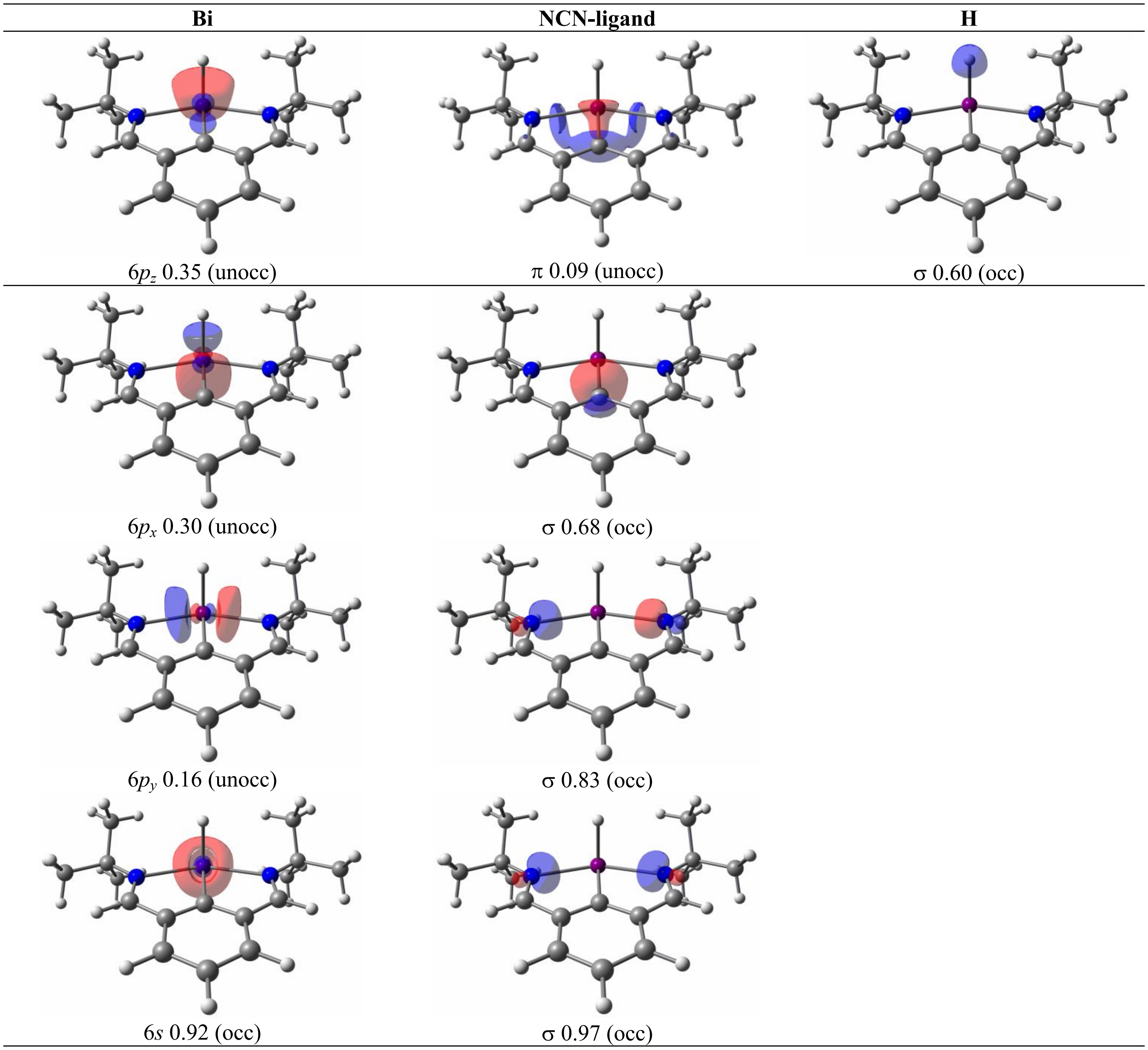

Figure S6. Valence Effective Fragment Orbitals (EFOs) at the B3LYP-D3(BJ)/def2-TZVPP level of theory of the Bi (left) and NCN-ligand (middle) and H (right) of 2-(H^+), together with symmetry, gross population and result of EOS analysis (occ/unocc). Isocontour value of 0.1 a.u., with exception of the 1s orbital on H (0.2 a.u.).

Bi	NNN ligand
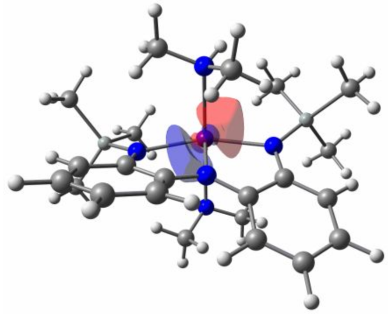	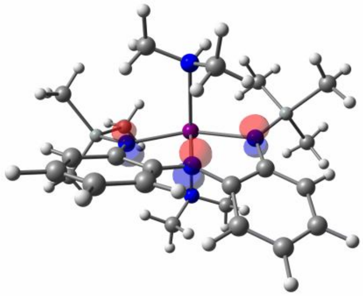
$6p_z$ 0.18 (unocc)	π 0.87 (occ)
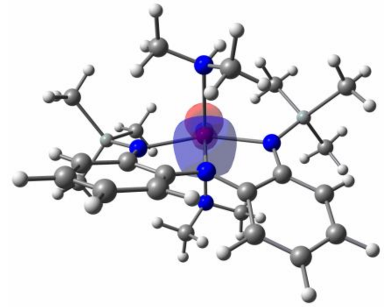	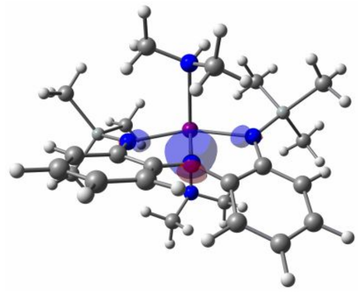
$6p_x$ 0.23 (unocc)	σ 0.77 (occ)
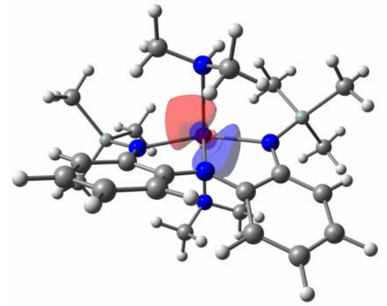	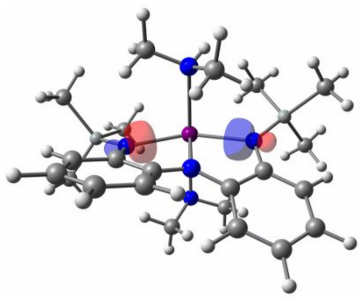
$6p_y$ 0.19 (unocc)	σ 0.80 (occ)
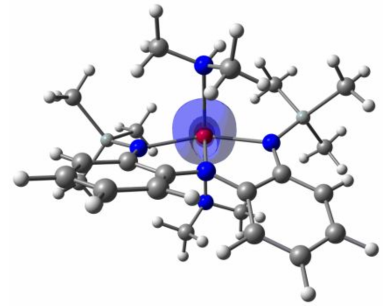	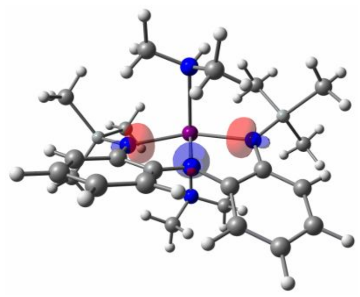
$6s$ 0.88 (occ)	σ 0.95 (occ)

Figure S7. Valence EFOs for the Bi (left) and NNN ligand (right) of 4-$(HNMe_2)_2$, together with symmetry, gross population and result of EOS analysis (occ/unocc). Isocontour value of 0.1 a.u.

Bi	NNN ligand
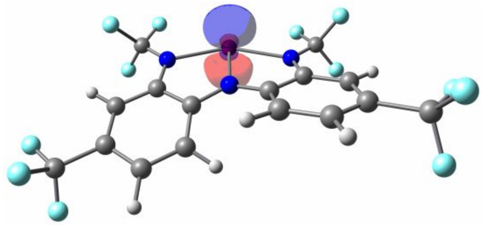<br>$6p_z$ 0.39 (unocc)	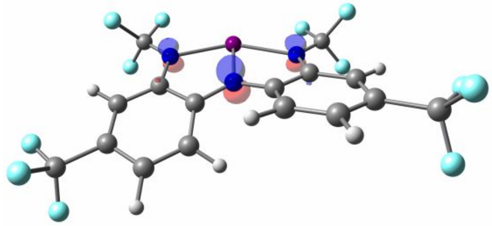<br>π 0.61 (occ)
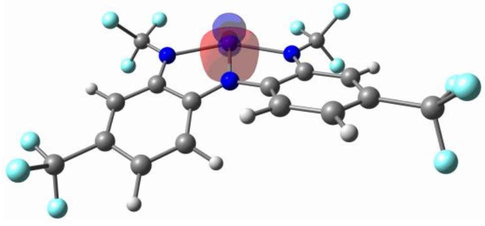<br>$6p_x$ 0.20 (unocc)	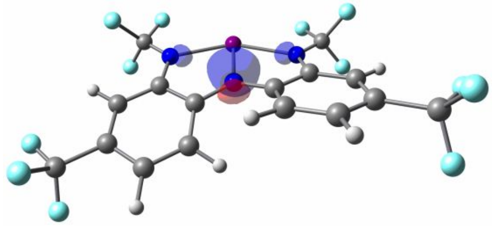<br>σ 0.80 (occ)
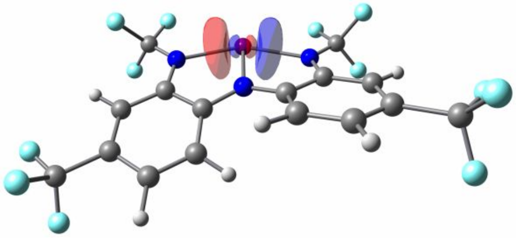<br>$6p_y$ 0.17 (unocc)	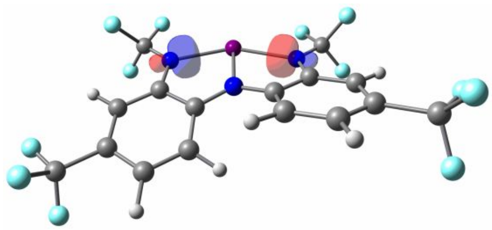<br>σ 0.83 (occ)
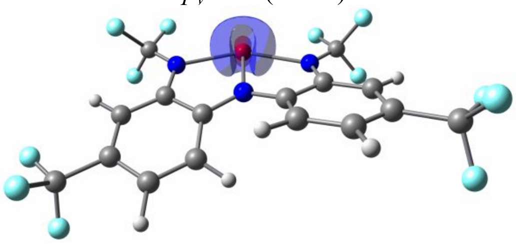<br>$6s$ 0.92 (occ)	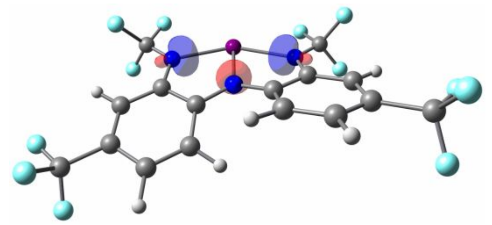<br>σ 0.96 (occ)

Figure S8. Valence EFOs for the Bi (left) and NNN ligand (right) of 5, together with symmetry, gross population and result of EOS analysis (occ/unocc). Isocontour value of 0.1 a.u.

Bi	NNN-ligand
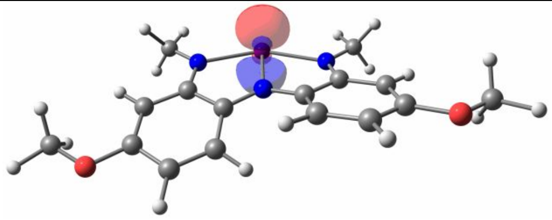	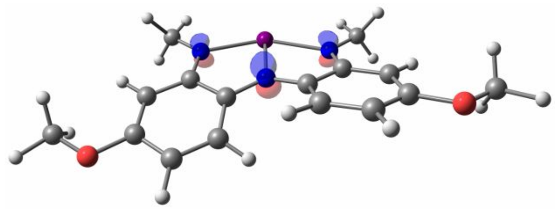
6p_z 0.48 (unocc)	π 0.52 (occ)
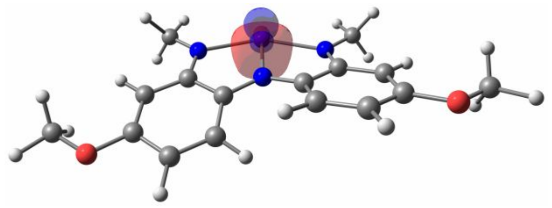	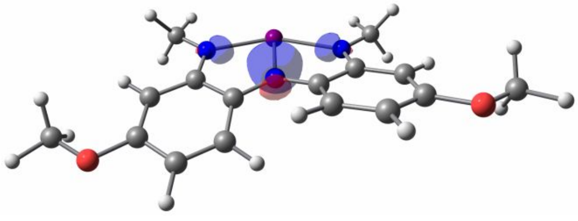
6p_x 0.21 (unocc)	σ 0.79 (occ)
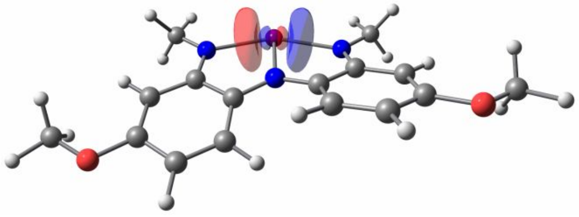	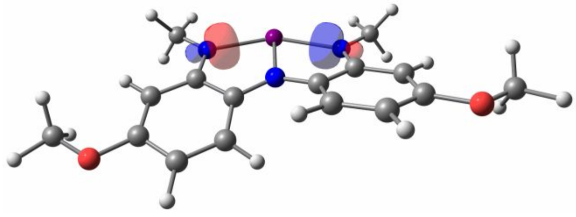
6p_y 0.17 (unocc)	σ 0.83 (occ)
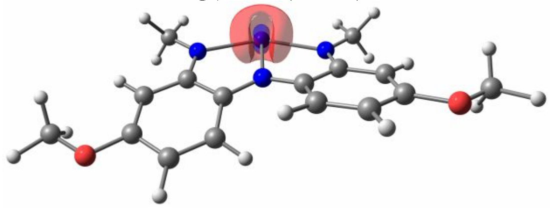	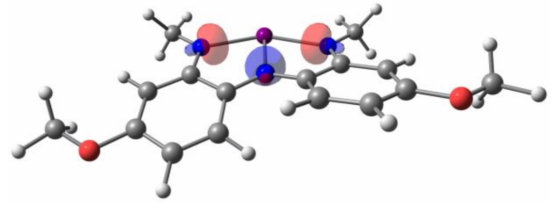
6s 0.91 (occ)	σ 0.96 (occ)

Figure S9. Valence EFOs for the Bi (left) and NNN ligand (right) of 6, together with symmetry, gross population and result of EOS analysis (occ/unocc). Isocontour value of 0.1 a.u.

Table S1. Electronic energy (in Hartree), Relative Electronic Energy (in kcal/mol), selected EFO gross populations and oxidation states along the Bi-W bond breaking potential energy surface for system **2-(W(CO)$_5$)**.

Bi-W Distance (Å)	Electronic Energy	ΔE	Occ. 6p_z (Bi)	Occ. π (NCN)	Occ. d(W(CO)$_5$)	Bi OS	NCN OS	W(CO)$_5$ OS	R (%)
3.011	-1582.3918	0.0	0.54	0.19	0.29	+1	-1	0	73.4
3.211	-1582.3887	2.0	0.54	0.20	0.27	+1	-1	0	74.8
3.411	-1582.3816	6.4	0.54	0.22	0.25	+1	-1	0	76.1
3.611	-1582.3727	12.0	0.55	0.23	0.23	+1	-1	0	78.6
3.811	-1582.3634	17.9	0.55	0.25	0.21	+1	-1	0	78.6
4.011	-1582.3562	22.4	0.56	0.26	0.18	+1	-1	0	79.4
4.211	-1582.3483	27.4	0.56	0.28	0.16	+1	-1	0	78.6
4.411	-1582.3413	31.8	0.57	0.29	0.15	+1	-1	0	77.5
4.611	-1582.3353	35.5	0.57	0.31	0.13	+1	-1	0	76.2
4.811	-1582.3302	38.7	0.57	0.32	0.11	+1	-1	0	75.2
5.011	-1582.3261	41.3	0.58	0.33	0.09	+1	-1	0	74.5

Table S2. Electronic energy (in Hartree), Relative Electronic Energy (in kcal/mol), selected EFO gross populations and oxidation states along the Bi-W bond breaking potential energy surface for system **4-(W(CO)$_5$)**.

Bi-W Distance (Å)	Electronic Energy	ΔE	Occ. 6p_z (Bi)	Occ. π (NCN)	Occ. d(W(CO)$_5$)	Bi OS	NNN OS	W(CO)$_5$ OS	R (%)
3.026	-2294.4126	0.0	0.48	0.31	0.23	+1	-1	0	65.6
3.226	-2294.4100	1.6	0.47	0.33	0.21	+1	-1	0	63.0
3.426	-2294.4040	5.4	0.47	0.36	0.18	+1	-1	0	59.9
3.626	-2294.3968	9.9	0.46	0.39	0.16	+1	-1	0	56.8
3.826	-2294.3894	14.6	0.46	0.42	0.13	+1	-1	0	53.7
4.026	-2294.3825	18.9	0.45	0.44	0.11	+1	-1	0	50.7
4.226	-2294.3765	22.7	0.45	0.47	0.09	+3	-3	0	52.3
4.426	-2294.3715	25.8	0.44	0.49	0.08	+3	-3	0	55.2
4.626	-2294.3675	28.3	0.43	0.51	0.06	+3	-3	0	58.0
4.826	-2294.3644	30.3	0.43	0.53	0.05	+3	-3	0	60.7
5.026	-2294.3620	31.8	0.42	0.55	0.04	+3	-3	0	62.8

Table S3. Electronic energy (in Hartree), Relative Electronic Energy (in kcal/mol), selected EFO gross populations and oxidation states along the Bi-W bond breaking potential energy surface for system **5-(W(CO)$_5$)**.

Bi-W Distance (Å)	Electronic Energy	ΔE	Occ. 6p_z (Bi)	Occ. π (NCN)	Occ. *d*(W(CO)$_5$)	Bi OS	NNN OS	W(CO)$_5$ OS	R (%)
3.019	-2825.5834	0.0	0.46	0.38	0.20	+1	-1	0	56.1
3.219	-2825.5810	1.5	0.46	0.41	0.17	+1	-1	0	53.2
3.419	-2825.5758	4.8	0.45	0.44	0.14	+3	-3	0	50.7
3.619	-2825.5701	8.4	0.44	0.47	0.11	+3	-3	0	54.2
3.819	-2825.5647	11.8	0.43	0.49	0.09	+3	-3	0	56.6
4.019	-2825.5602	14.6	0.42	0.51	0.07	+3	-3	0	59.0
4.219	-2825.5567	16.8	0.42	0.53	0.06	+3	-3	0	62.0
4.419	-2825.5540	18.5	0.41	0.55	0.05	+3	-3	0	64.2
4.619	-2825.5519	19.8	0.40	0.57	0.04	+3	-3	0	66.2
4.819	-2825.5503	20.8	0.40	0.58	0.03	+3	-3	0	68.2
5.019	-2825.5490	21.6	0.39	0.59	0.02	+3	-3	0	69.7

Table S4. Electronic energy (in Hartree), Relative Electronic Energy (in kcal/mol), selected EFO gross populations and oxidation states along the Bi-W bond breaking potential energy surface for system **6-(W(CO)$_5$)**.

Bi-W Distance (Å)	Electronic Energy	ΔE	Occ. 6p_z (Bi)	Occ. π (NCN)	Occ. *d*(W(CO)$_5$)	Bi OS	NNN OS	W(CO)$_5$ OS	R (%)
2.972	-1784.6142	0.0	0.51	0.25	0.27	+1	-1	0	73.0
3.172	-1784.6112	1.9	0.51	0.27	0.24	+1	-1	0	72.4
3.372	-1784.6049	5.8	0.51	0.29	0.21	+1	-1	0	70.0
3.572	-1784.5973	10.7	0.51	0.32	0.19	+1	-1	0	67.3
3.772	-1784.5893	15.7	0.51	0.34	0.16	+1	-1	0	65.0
3.972	-1784.5821	20.2	0.50	0.37	0.14	+1	-1	0	62.3
4.172	-1784.5759	24.1	0.49	0.40	0.12	+1	-1	0	59.3
4.372	-1784.5708	27.3	0.49	0.42	0.10	+1	-1	0	56.6
4.572	-1784.5665	30.0	0.48	0.44	0.08	+1	-1	0	54.1
4.772	-1784.5630	32.2	0.48	0.46	0.07	+1	-1	0	51.9
4.972	-1784.5601	34.0	0.47	0.48	0.06	+3	-3	0	50.2

Table S5. Selected EFO gross populations and oxidation states of systems **2** and **4** evaluated with different KS-DFT functionals and basis sets.[a]

	Compound **2**					Compound **4**				
Funct/Basis set	Occ. 6p_z (Bi)	Occ. π (NCN)	Bi OS	NCN OS	R (%)	Occ. 6p_z (Bi)	Occ. π (NNN)	Bi OS	NNN OS	R (%)
BP86/def2-SVP	0.57	0.43	+1	-1	64.4	0.44	0.56	+3	-3	62.3
BP86/def2-TZVP	0.57	0.43	+1	-1	63.9	0.44	0.57	+3	-3	63.1
BP86/def2-TZVPP	0.57	0.43	+1	-1	63.8	0.43	0.57	+3	-3	63.2
B3LYP/def2-SVP	0.60	0.41	+1	-1	68.9	0.43	0.57	+3	-3	64.1
B3LYP/def2-TZVP	0.59	0.41	+1	-1	68.2	0.43	0.58	+3	-3	64.9
B3LYP/def2-TZVPP	0.59	0.41	+1	-1	68.1	0.43	0.58	+3	-3	65.0
PBE0/def2-SVP	0.59	0.41	+1	-1	68.8	0.42	0.58	+3	-3	66.4
PBE0/def2-TZVP	0.59	0.41	+1	-1	68.4	0.42	0.59	+3	-3	66.9
PBE0/def2-TZVPP	0.59	0.41	+1	-1	68.4	0.42	0.59	+3	-3	67.0
M062X/def2-SVP	0.67	0.37	+1	-1	75.8	0.39	0.61	+3	-3	71.1
M062X/def2-TZVP	0.62	0.38	+1	-1	74.6	0.37	0.63	+3	-3	75.2
M062X/def2-TZVPP	0.62	0.38	+1	-1	74.5	0.37	0.63	+3	-3	75.4
wB97xD/def2-SVP	0.62	0.39	+1	-1	72.9	0.40	0.60	+3	-3	69.9
wB97xD/def2-TZVP	0.61	0.39	+1	-1	72.7	0.40	0.60	+3	-3	70.3
wB97xD/def2-TZVPP	0.61	0.39	+1	-1	72.6	0.40	0.60	+3	-3	70.4

[a] All calculations have been performed on the B3LYP-D3(BJ)/def2-TZVPP optimized geometries.

Table S6. Aromaticity indices (NICS and PDI) for the selected molecular systems studied at the B3LYP-D3(BJ)/def2-TZVPP level of theory.

Mol.	PDI[1]	PDI[2]	|PDI|	NICS (1,-1)[1]	NICS (1,-1)[1]	NICS (1,-1)[2]	NICS (1,-1)[2]	NICS (|1|)	NICS (0)[1]	NICS (0)[2]	NICS (|0|)
**1**	0.070	-	0.070	8.6	8.6	-	-	8.6	6.5	-	6.5
**1-(H^+)**	0.085	-	0.085	9.1	8.9	-	-	9.0	6.5	-	6.5
**1-($W(CO)_5$)**	0.079	-	0.079	9.2	8.9	-	-	9.0	6.8	-	6.8
**2**	0.068	-	0.068	8.9	8.9	-	-	8.9	6.8	-	6.8
**2-(H^+)**	0.083	-	0.083	9.0	9.3	-	-	9.1	6.6	-	6.6
**2-($W(CO)_5$)**	0.077	-	0.077	9.5	9.7	-	-	9.6	7.4	-	7.4
**3**	0.066	-	0.066	8.0	8.0	-	-	8.0	5.9	-	5.9
**3-(H^+)**	0.077	-	0.077	8.4	8.2	-	-	8.3	5.8	-	5.8
**3-($W(CO)_5$)**	0.076	-	0.076	8.6	9.1	-	-	8.9	6.6	-	6.6
**4**	0.068	0.068	0.068	10.0	8.2	8.2	10.0	9.1	7.7	7.7	7.7
**4-(H^+)**	0.053	0.052	0.053	3.0	4.4	4.4	3.5	3.8	0.3	0.6	0.4
**4-($W(CO)_5$)**	0.058	0.059	0.059	6.1	7.5	8.0	5.9	6.9	4.1	4.6	4.4
**5**	0.069	0.069	0.069	8.6	10.1	10.1	8.6	9.3	8.7	8.7	8.7
**5-(H^+)**	0.051	0.053	0.052	2.9	4.3	4.5	3.6	3.8	0.8	1.3	1.0
**5-($W(CO)_5$)**	0.060	0.062	0.061	8.5	6.9	6.9	8.9	7.8	5.9	6.4	6.2
**6**	0.057	0.057	0.057	9.4	7.6	7.6	9.4	8.5	7.9	7.9	7.9
**6-(H^+)**	0.041	0.043	0.042	2.8	4.1	4.2	3.3	3.6	1.2	1.5	1.3
**6-($W(CO)_5$)**	0.048	0.048	0.048	6.5	5.2	5.0	7.0	5.9	4.1	4.4	4.3

Table S7. EOS analysis on Bi-based molecular systems at the B3LYP-D3(BJ)/def2-TZVPP level of theory. L = Chelating ligand.

ref.	Molecular Structure	Ref OS	Bi OS	L OS	R(%)	LO EFO occ. (Frag.)	FU EFO occ. (Frag.)
	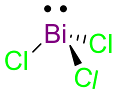	+3	3	-1	100	0.77 (Cl)	0.27 (Bi)
	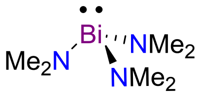	+3	+3	-1	100	0.78 (NMe_2)	0.28 (Bi)
S[1]	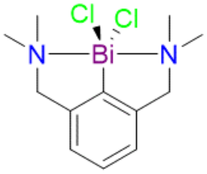	+3	+3	-1	87.9	0.68 (L)	0.31 (Bi)
S[2]	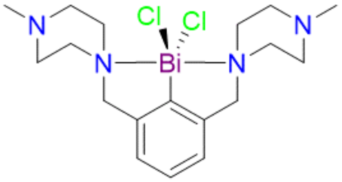	+3	+3	-1	87.0	0.68 (L)	0.31 (Bi)
S[2]	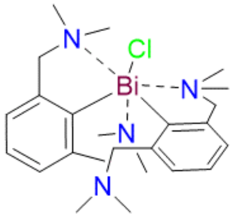	+3	+3	-1	87.6	0.69 (L)	0.32 (Bi)
S[3]	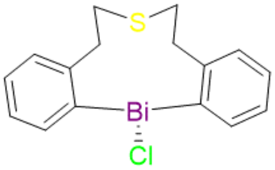	+3	+3	-2	76.7	0.63 (L)	0.37 (Bi)
S[3]	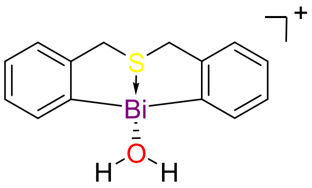	+3	+3	-2	79.6	0.64 (L)	0.35 (Bi)
S[4]	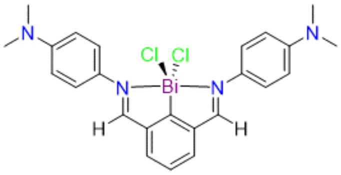	+3	+3	-1	91.4	0.70 (L)	0.29 (Bi)

S[4]	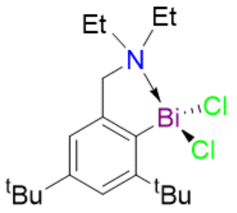	+3	+3	-1	86.0	0.67 (L)	0.32 (Bi)
S[4]	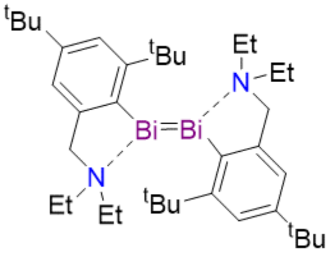	+1	+1	-1	58.2	0.47 (Bi_1/Bi_2)	0.39 (Bi_2/Bi_1)
S[4]	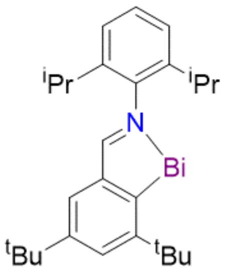	+1	+1	-1	66.2	0.58 (Bi)	0.42 (L)
S[4]	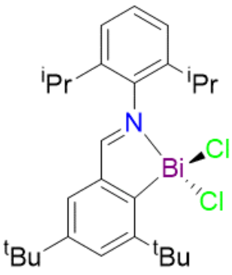	+3	+3	-1	88.6	0.69 (L)	0.31 (Bi)
S[5]	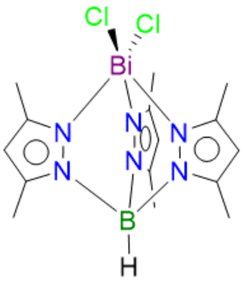	+3	+3	-1	100.0	0.83 (L)	0.20 (Bi)
S[5]	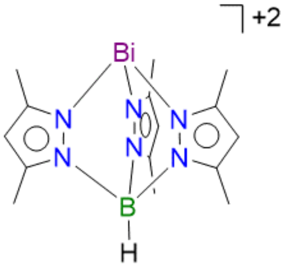	+3	+3	-1	100.0	0.77 (L)	0.23 (Bi)

S[6]	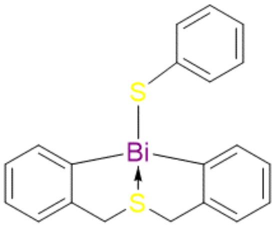	+3	+3	-2	82.8	0.66 (L)	0.34 (Bi)
S[7]	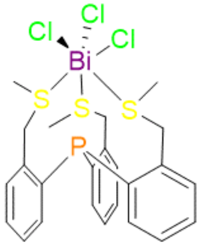	+3	+3	-1	100.0	0.80 (Cl)	0.23 (Bi)
S[8]	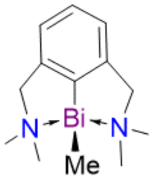	+3	+3	-1	83.2	0.65 (CH_3)	0.33 (Bi)
S[9]	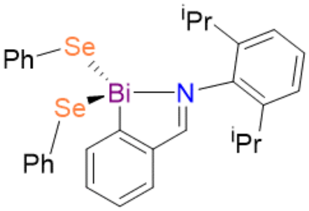	+3	+3	-1	82.7	0.66 (Se)	0.34 (Bi)
S[9]	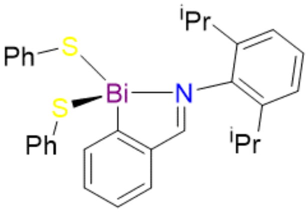	+3	+3	-1	87.9	0.69 (L)	0.32 (Bi)

Table S8. NBO results of **1** at B3LYP-D3(BJ)/def2-TZVPP level of theory. Total non-Lewis (2.07%).

**Orbital**	**Occ**	**Contribution from atoms to orbitals**	**Atomic orbitals**
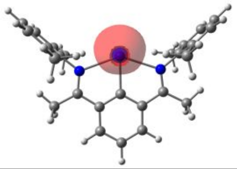	1.99	Bi (LP)	Bi: s(89.76%)p (10.23%)d (0.01%) f (0.00%)
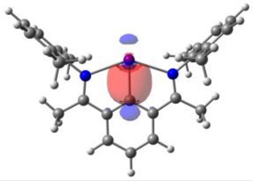	1.95	Bi (30.06%)- C (69.94%)	Bi: s(10.65%)p (89.18%)d (0.07%) f (0.10%)<br>C: s(28.19%)p (71.70%)d (0.09%) f (0.03%)
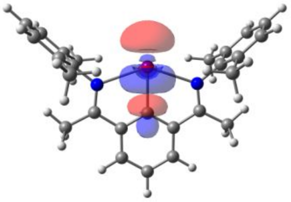	0.05	Bi(69.94%)- C (30.06%)	Bi: s(10.65%)p (89.18%) d (0.07%) f (0.10%)<br>C: s(28.19%)p (71.70%)d (0.09%)
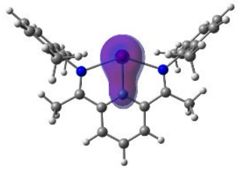	1.83	Bi (67.28%) – C (32.72%)	Bi :s(0.00%)p (99.96%)d (0.02%) f (0.02%)<br>C : s(0.00%)p (99.91%)d (0.04%) f (0.05%)
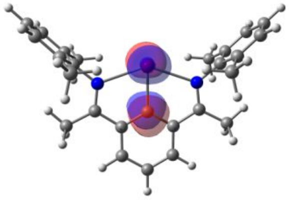	0.62	Bi (32.72%) – C (67.28%)	Bi: s(0.00%)p (99.96%)d (0.02%) f (0.02%)<br>C: s(0.00%)p (99.91%)d (0.04%) f (0.05%)
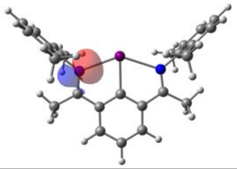	1.74	N (LP)	N: s(28.23%)p (71.74%)d (0.02%) f (0.01%)
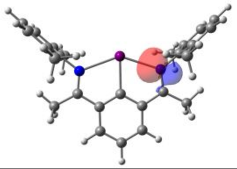	1.74	N(LP)	N:s(28.23%)p (71.74%)d (0.02%) f (0.01%)
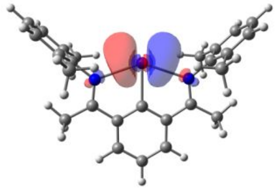	0.32	Bi (LV)	Bi: s(0.00%)p (99.83%)d (0.03%) f (0.14%)

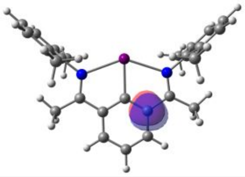	1.06	C(LP)	C: s(0.00%)p (99.94%)d (0.00%) f (0.06%)
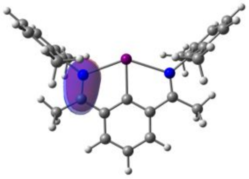	1.93	N (65.84%) – C(34.16%)	N: s(0.00%)p (99.83%)d (0.14%) f (0.02%)<br>C: s(0.00%)p (99.85%)d (0.11%) f (0.04%)
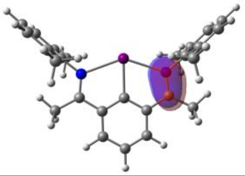	1.94	N (65.84%) – C (34.16%)	N: s(0.00%)p (99.83%)d (0.14%) f (0.02%)<br>C: s(0.00%)p (99.85%)d (0.11%) f (0.04%)
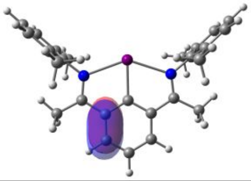	1.63	C (53.94%) – C(46.06%)	C: s(0.00%)p (99.93%)d (0.01%) f (0.05%)<br>C: s(0.00%)p (99.93%)d (0.03%) f (0.04%)
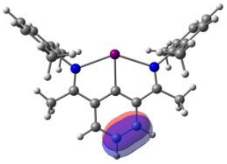	1.66	C(46.47%) – C(53.53%)	C: s(0.00%)p (99.93%)d (0.03%) f (0.04%)<br>C: s(0.00%)p (99.94%)d (0.02%) f (0.04%)

Table S9. NBO results of **2** at B3LYP-D3(BJ)/def2-TZVPP level of theory. Total non-Lewis (1.50%).

Orbital	Occ	Contribution from atoms to orbitals	Atomic orbitals
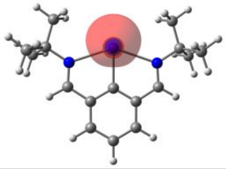	1.99	Bi (LP)	Bi:s(89.89%)p (10.11%)d (0.00%) f (0.00%)
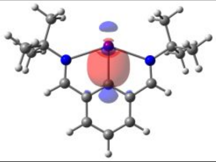	1.95	Bi (30.13%)- C (69.87%)	Bi: s(10.61%)p (89.23%)d (0.05%) f (0.10%)<br>C : s(28.55%)p (71.33%)d (0.10%) f (0.03%)
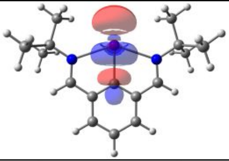	0.05	Bi (69.87%) – C (30.13%)	Bi: s(10.61%)p (89.23%)d (0.05%) f (0.10%)<br>C: s(28.55%)p (71.33%)d (0.10%) f (0.03%)
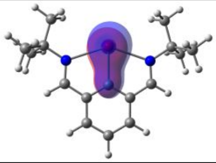	1.82	Bi (65.39%) – C (34.61%)	Bi : s(0.00%)p (99.96%)d (0.02%) f (0.03%)<br>C: s(0.00%)p (99.91%)d (0.04%) f (0.05%)
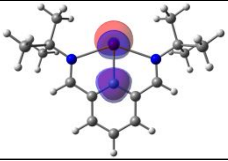	0.62	Bi (34.61%) – C (65.39%)	Bi: s(0.00%)p (99.96%)d (0.02%) f (0.03%)<br>C: s(0.00%)p (99.91%)d (0.04%) f (0.05%)
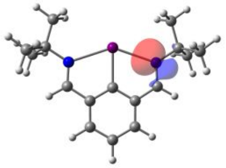	1.77	N (LP)	N: s(32.85%)p (67.12%)d (0.03%) f (0.00%)
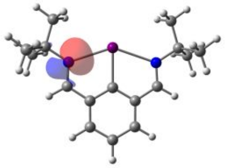	1.77	N (LP)	N: s(32.85%)p (67.12%)d (0.03%) f (0.00%)
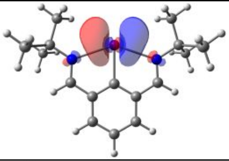	0.33	LV(Bi)	Bi : s(0.00%)p (99.83%)d (0.03%) f (0.14%)
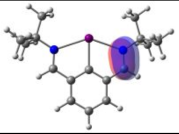	1.94	N (63.72%) – C (36.28%)	N :s(0.00%)p 1.00(99.84%)d (0.14%) f (0.02%)<br>C: s(0.00%)p (99.86%)d (0.10%) f (0.04%
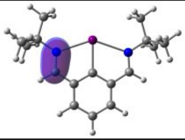	1.94	N (63.72%) – C (36.28%)	N : s(0.00%)p (99.84%)d (0.14%) f (0.02%)<br>C: s(0.00%)p (99.86%)d (0.10%) f (0.04%)

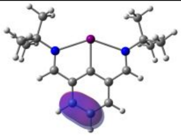	1.66	C(46.20%) – C (53.80%)	C: s(0.00%)p (99.93%)d (0.03%) f (0.04%)<br>C : s(0.00%)p (99.93%)d (0.02%) f (0.04%)
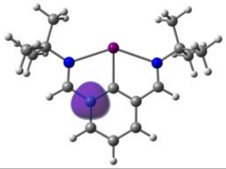	1.05	C (LP)	C: s(0.00%)p (99.94%)d (0.00%)<br>f 0.00(0.05%)

Table S10. NBO results of **3** at B3LYP-D3(BJ)/def2-TZVPP level of theory. Total non-Lewis (2.19%).

Orbital	Occ	Contribution from atoms to orbitals	Atomic orbitals
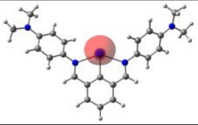	1.99	Bi (LP)	Bi: s(89.65%)p (10.34%)d 0.00(0.00%) f (0.00%)
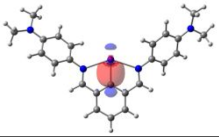	1.95	Bi (30.58%) – C (69.42)	Bi: s(10.79%)p (89.05%)d (0.06%) f (0.10%)<br>C: s(28.21%)p (71.66%)d (0.10%) f (0.03%)
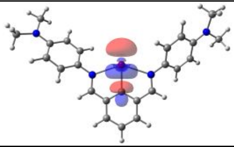	0.05	Bi (69.42%) – C (30.58%)	Bi : s(10.79%)p (89.05%)d (0.06%) f (0.10%)<br>C: s(28.21%)p (71.66%)d (0.10%) f (0.03%)
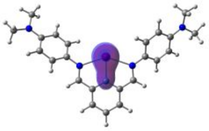	1.82	Bi (64.52%) – C (35.48%)	Bi: s(0.00%)p (99.96%)d (0.02%) f (0.03%)<br>C: s(0.00%)p (99.91%)d (0.04%) f (0.05%)
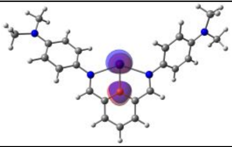	0.61	Bi(35.48%) – C(64.52%)	Bi: s(0.00%)p (99.96%)d (0.02%) f (0.03%)<br>C: s(0.00%)p (99.91%)d (0.04%) f (0.05%)
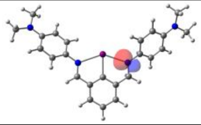	1.76	Bi (LP)	Bi: s(29.65%)p (70.32%)d (0.03%) f (0.00%)
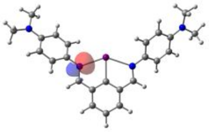	1.76	Bi (LP)	Bi: s(29.66%)p (70.31%)d (0.03%) f (0.00%)
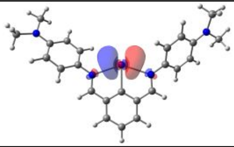	0.33	Bi (LV)	Bi: s(0.00%)p (99.80%)d (0.04%) f 0.00(0.16%)
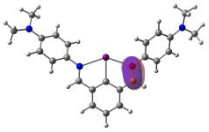	1.91	N(64.02%) – C (35.98%)	N: s(0.11%)p (99.74%)d (0.13%) f (0.02%)<br>C: s(0.21%)p (99.65%)d (0.09%) f (0.04%)
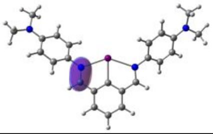	1.91	N(64.01%) –C (35.99%)	N: s(0.11%)p (99.74%)d 1.17(0.13%) f (0.02%)<br>C: s(0.21%)p (99.65%)d (0.09%) f (0.04%)
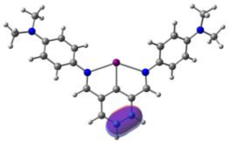	1.67	C(46.40%) – C (53.60%)	C: s(0.00%)p (99.93%)d (0.03%) f (0.04%)<br>C: s(0.00%)p (99.93%)d (0.02%) f (0.04%)
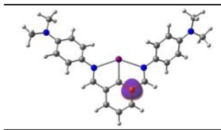	1.05	Bi (C)	C: s(0.00%)p (99.94%)d 0.00(0.00%) f (0.06%)

Table S11. NBO results of **4** at B3LYP-D3(BJ)/def2-TZVPP level of theory. Total non-Lewis (1.84%).

**Orbital**	**Occ**	**Contribution from atoms to orbitals**	**Atomic orbitals**
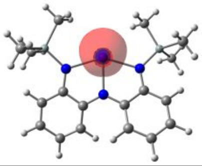	1.98	Bi (LP)	Bi: s(92.69%)p (7.30%)d (0.01%) f (0.00%)
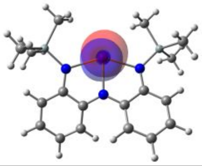	1.00	Bi (LP)	Bi: s(0.00%)p (99.96%)d (0.01%) f (0.02%)
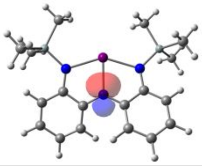	1.66	N (LP)	N: s(28.01%)p (71.96%)d (0.02%) f (0.01%)
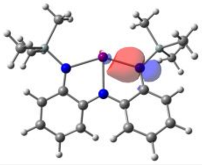	1.93	Bi (11.94%) – N (88.06%)	Bi : s(6.43%)p (92.74%)d (0.44%) f (0.38%)<br>N: s(22.50%)p (77.49%)d (0.01%) f (0.01%)

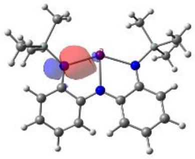	1.93	Bi(11.94%) – N(88.06%)	Bi : s(6.43%)p (92.74%)d (0.44%) f (0.38%)<br>N: s(22.50%)p (77.49%)d (0.01%) f (0.01%)
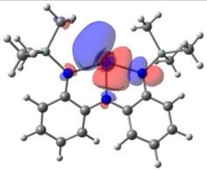	0.18	Bi (88.06%) – N(11.94%)	Bi : s(6.43%)p(92.74%)d (0.44%) f (0.38%)<br>N: s(22.50%)p (77.49%)d (0.01%) f (0.01%)
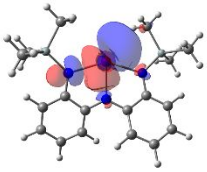	0.19	Bi (88.06%) – N(11.94%)	Bi : s(6.43%)p (92.74%)d (0.44%) f (0.38%)<br>N: s(22.50%)p (77.49%)d (0.01%) f (0.01%)
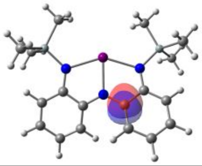	1.03	C (LP)	C: s(0.01%)p (99.95%)d (0.00%) f (0.05%)
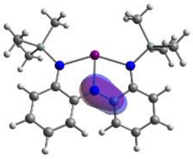	1.76	N(72.34%) – C (27.66%)	N: s(35.91%)p (63.88%)d 0.01(0.19%) f (0.02%)<br>C: s(31.16%)p (68.73%)d (0.08%) f (0.04%)
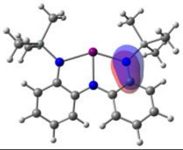	1.88	N(75.24%) – C (24.76%)	N: s(0.01%)p (99.93%)d (0.04%) f (0.02%)<br>C: s(0.00%)p (99.86%)d (0.09%) f (0.05%)
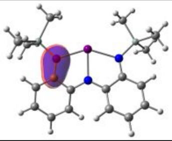	1.88	N(75.24%) – C (24.76%)	N: s(0.01%)p (99.93%)d (0.04%) f(0.02%)<br>C: s(0.00%)p (99.86%)d (0.09%) f (0.05%)
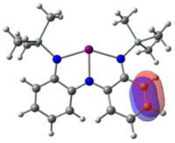	1.75	C(51.92%) – C (48.08%)	C: s(0.01%)p (99.92%)d (0.02%) f (0.04%)<br>C: s(0.02%)p (99.91%)d (0.03%) f (0.04%)
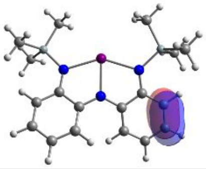	1.75	C(51.92%) – C(48.08%)	C: s(0.01%)p (99.92%)d (0.02%) f (0.04%)<br>C: s(0.02%)p (99.91%)d (0.03%) f (0.04%)
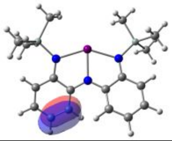	1.74	C(50.01%)- C(49.99%)	C: s(0.00%)p (99.93%)d (0.02%) f (0.04%)<br>C: s(0.00%)p (99.93%)d (0.03%) f (0.04%)
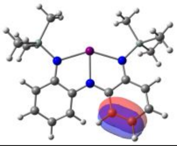	1.74	C(50.01%) – C(49.99%)	C: s(0.00%)p (99.93%)d (0.02%) f (0.04%)<br>C: s(0.00%)p (99.93%)d (0.03%) f (0.04%)

Table S12. NBO results of **5** at B3LYP-D3(BJ)/def2-TZVPP level of theory. Total non-Lewis (1.89%).

**Orbital**	**Occ**	**Contribution from atoms to orbitals**	**Atomic orbitals**
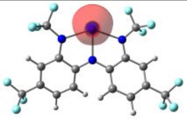	1.99	Bi(LP)	Bi: s(93.58%)p (6.41%)d (0.01%) f (0.00%)
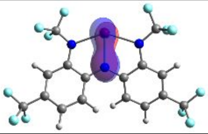	1.78	Bi (23.89%) – N (76.11%)	Bi: s(0.00%)p (99.70%)d (0.16%) f (0.14%)<br>N: s(0.00%)p (99.98%)d (0.00%) f (0.02%)
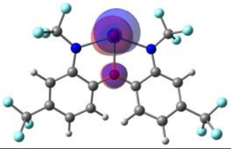	0.62	Bi(76.11%) – N(23.89%)	Bi: s(0.00%)p (99.70%)d (0.16%) f (0.14%)<br>N: s(0.00%)p (99.98%)d (0.00%) f (0.02%)
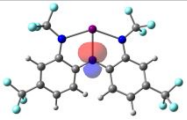	1.66	N(LP)	N: s(29.68%)p (70.29%)d(0.02%) f (0.01%)
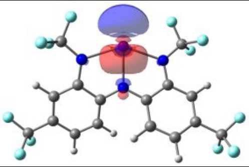	0.41	Bi(LV)	Bi: s(7.29%)p (92.48%)d (0.15%) f (0.08%)
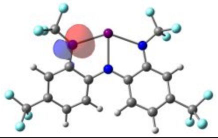	1.73	N(LP)	N: s(33.92%)p (66.05%)d (0.02%) f (0.01%)
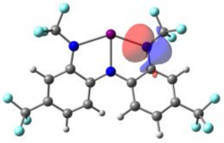	1.73	N(LP)	N: s(33.92%)p (66.05%)d (0.02%) f (0.01%)
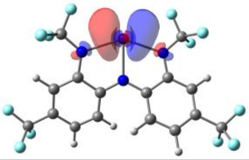	0.35	Bi(LV)	Bi: s(0.00%)p (99.80%)d (0.03%) f (0.17%)
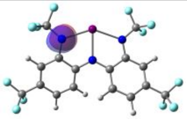	1.60	N(LP)	N: s(0.46%)p (99.48%)d (0.03%) f (0.03%)
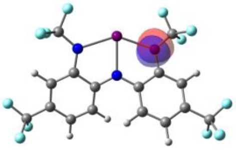	1.60	N(LP)	N: s(0.46%)p (99.48%)d (0.03%) f (0.03%)
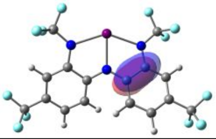	1.53	C(47.87%) – C (52.13%)	C: s(0.00%)p (99.92%)d (0.02%) f (0.05%)<br>C: s(0.00%)p (99.93%)d (0.02%) f (0.05%)

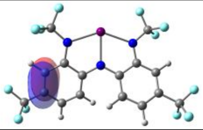	1.73	C (48.71%)- C(51.29%)	C: s(0.01%)p (99.91%)d (0.04%) f (0.05%)<br>C: s(0.03%)p (99.90%)d (0.02%) f (0.05%)
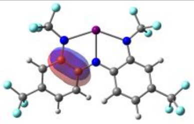	1.53	C(47.87%) – C(52.13%)	C: s(0.00%)p (99.92%)d (0.02%) f (0.05%)<br>C: s(0.00%)p (99.93%)d (0.02%) f (0.05%)
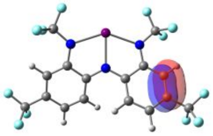	1.73	C(48.71%) – C (51.29%)	C: s(0.01%)p (99.91%)d (0.04%) f (0.05%)<br>C: s(0.03%)p (99.90%)d (0.02%) f (0.05%)
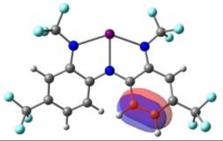	1.73	C (50.84%) – C(49.16%)	C: s(0.00%)p (99.92%)d (0.03%) f (0.04%)<br>C: s(0.00%)p (99.92%)d (0.03%) f (0.05%)
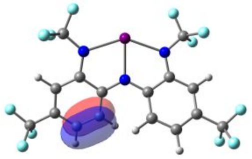	1.73	C(50.84%) – C (49.16%)	C: s(0.00%)p (99.92%)d (0.03%) f (0.04%)<br>C: s(0.00%)p (99.92%)d (0.03%) f (0.05%)

Table S13. NBO results of **6** at B3LYP-D3(BJ)/def2-TZVPP level of theory. Total non-Lewis (2.10%).

Orbital	Occ	Contribution from atoms to orbitals	Atomic orbitals
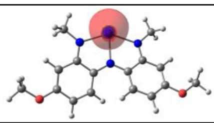	1.99	Bi(LP)	Bi: s(91.83%)p (8.17%)d (0.00%) f (0.00%)
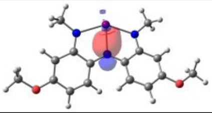	1.93	Bi(15.88%) – N(84.12%)	Bi: s(7.86%)p (91.48%)d (0.25%) f (0.41%)<br>N: s(24.34%)p (75.64%)d (0.02%) f (0.01%)
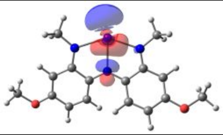	0.16	Bi(84.12%) – N(15.88%)	Bi: s(7.86%)p (91.48%)d (0.25%) f (0.41%)<br>C: s(24.34%)p (75.64%)d (0.02%) f (0.01%)
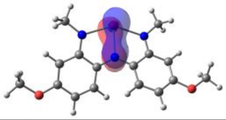	1.80	Bi (32.45%) – N (67.55%)	Bi: s(0.00%)p (99.85%)d (0.06%) f (0.09%)<br>N: s(0.00%)p (99.97%)d (0.00%) f (0.02%)
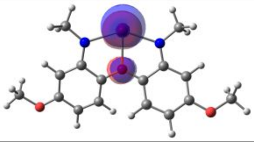	0.80	Bi(67.55%) – N(32.45%)	Bi: s(0.00%)p (99.85%)d (0.06%) f (0.09%)<br>N:s(0.00%)p (99.97%)d (0.00%) f (0.02%)
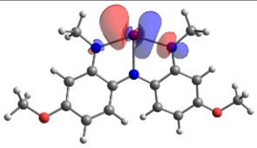	0.15	Bi(87.38%) – N(12.62%)	Bi: s(1.21%)p (98.02%)d (0.29%) f (0.47%)<br>N: s(27.02%)p (72.96%)d (0.01%) f (0.01%)
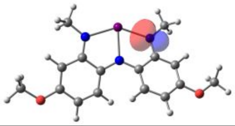	1.67	N(LP)	N: s(20.05%)p (79.94%)d (0.01%) f (0.01%)
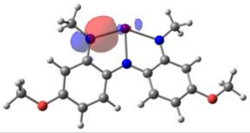	1.93	Bi (12.62%) – N (87.38%)	Bi: s(1.21%)p (98.02%)d (0.29%) f (0.47%)<br>N: s(27.02%)p (72.96%)d (0.01%) f (0.01%)
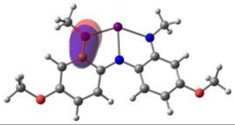	1.89	N(72.64%) – C (27.36%)	N: s(0.05%)p99.99(99.88%)d (0.05%) f (0.02%)<br>C: s(0.01%)p (99.85%)d (0.09%) f (0.05%)
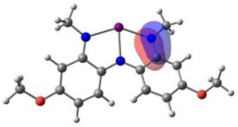	1.89	N(72.85%) – C(27.15%)	N: s(0.28%)p (99.65%)d (0.05%) f (0.02%)<br>C: s(0.01%)p (99.85%)d (0.09%) f (0.05%)
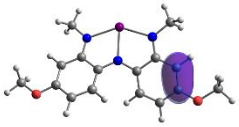	1.76	C(57.11%) – C(42.89%)	C: s(0.01%)p (99.91%)d (0.03%) f (0.05%)<br>C: s(0.01%)p (99.90%)d (0.05%) f (0.04%)
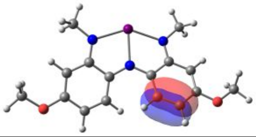	1.77	C(48.24%) – C (51.76%)	C: s(0.00%)p (99.93%)d (0.03%) f (0.04%)<br>C: s(0.00%)p (99.92%)d (0.03%) f (0.05%)

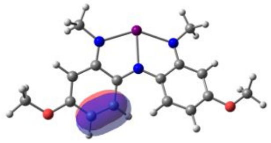	1.77	C(48.24%) – C(51.76%)	C: s(0.00%)p (99.93%)d (0.03%) f (0.04%)<br>C: s(0.00%)p (99.92%)d (0.03%) f (0.05%)
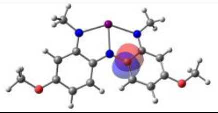	1.05	C(LP)	C: s(0.00%)p (99.95%)d (0.00%) f (0.05%)
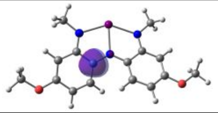	1.05	C(LP)	C: s(0.00%)p (99.95%)d (0.00%) f (0.05%)

Table S14. Lewis structure-constrained NBO results of **3** at B3LYP-D3(BJ)/def2-TZVPP level of theory. Total non-Lewis (2.35%).

Orbital	Occ	Contribution from atoms to orbitals	Atomic orbitals
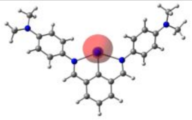	1.99	Bi(LP)	Bi: s(89.65%)p (10.34%)d (0.00%) f(0.00%)
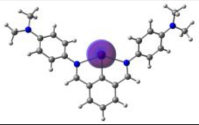	1.39	Bi(LP)	Bi: s(0.00%)p (99.98%)d(0.01%) f (0.02%)
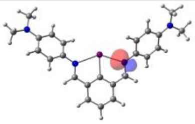	1.76	N(LP)	N: s(29.65%)p (70.32%)d (0.03%) f (0.00%)
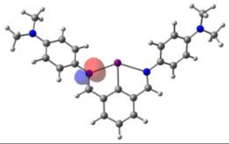	1.76	N(LP)	N: s(29.66%)p (70.31%)d (0.03%) f (0.00%)
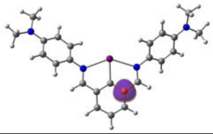	1.05	C(LP)	C: s(0.00%)p(99.94%)d(0.00%) f (0.06%)
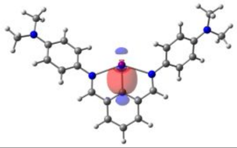	1.95	Bi(30.58%) – C (69.42%)	Bi: s(10.79%)p (89.05%)d (0.06%) f(0.10%)<br>C: s(28.21%)p (71.66%)d (0.10%) f (0.03%)
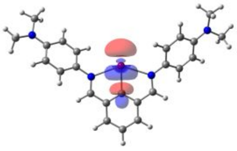	0.05	Bi (69.42%) – C(30.58%)	Bi: s(10.79%)p(89.05%)d (0.06%) f (0.10%)<br>C: s(28.21%)p (71.66%)d (0.10%) f (0.03%)
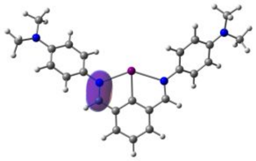	1.91	N(64.01%)- C(35.99%)	N: s(0.11%)p(99.74%)d (0.13%) f (0.02%)<br>C: s(0.21%)p(99.65%)d (0.09%) f (0.04%)
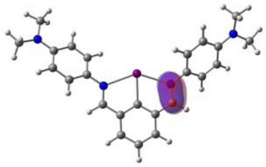	1.91	N(64.01%)- C(35.98%)	N: s(0.11%)p(99.74%)d (0.13%) f (0.02%)<br>C: s(0.21%)p(99.65%)d (0.09%) f(0.04%)
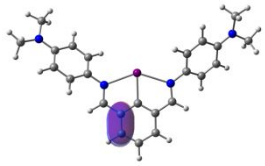	1.62	N(53.57%) –C(46.43%)	N: s(0.00%)p (99.94%)d (0.01%) f (0.05%)<br>C: s(0.00%)p (99.93%)d (0.03%) f (0.04%)

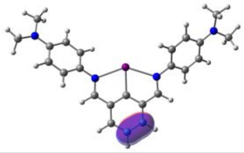	1.67	C(46.40%) –C(53.60%)	C: s(0.00%)p (99.93%)d (0.03%) f (0.04%)<br>C: s(0.00%)p (99.93%)d (0.02%) f (0.04%)
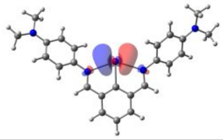	0.33	Bi(LV)	Bi: s(0.00%)p (99.80%)d (0.04%) f (0.16%)
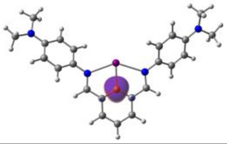	1.04	C(LV)	C: s(0.00%)p (99.95%)d(0.01%) f (0.04%)

Table S15. Lewis structure-constrained NBO results of **4** at B3LYP-D3(BJ)/def2-TZVPP level of theory. Total non-Lewis (1.74%).

Orbital	Occ	Contribution from atoms to orbitals	Atomic orbitals
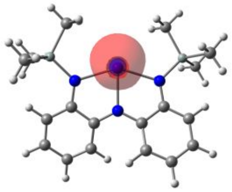	1.98	Bi(LP)	Bi: s(92.69%)p (7.30%)d (0.01%) f (0.00%)
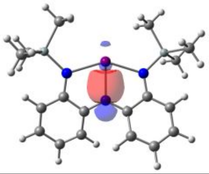	1.95	Bi (16.19%) – N (83.81%)	Bi: s(7.93%)p(91.66%)d (0.15%)f (0.26%)<br>N: s(24.96%)p (75.01%)d (0.02%) f (0.01%)
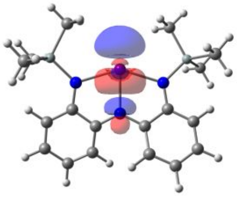	0.13	Bi(83.81%) – N(16.19%)	Bi: s(7.93%)p(91.66%)d (0.15%)f(0.26%)<br>N: s(24.96%)p (75.01%)d (0.02%)f (0.01%)
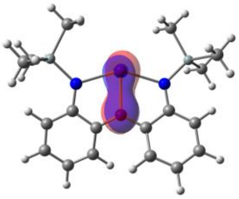	1.78	Bi (27.16%) – N (72.84%)	Bi: s(0.00%)p (99.79%)d (0.09%) f(0.12%)<br>N: s(0.00%)p (99.97%)d (0.00%) f (0.02%)
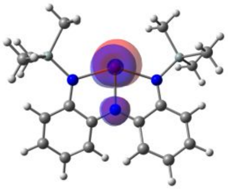	0.71	Bi(72.84%) – N(27.16%)	Bi: s(0.00%)p (99.79%)d (0.09%) f (0.12%)<br>N: s(0.00%)p (99.97%)d (0.00%)f (0.02%)
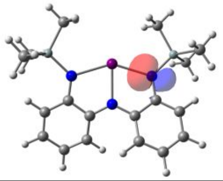	1.72	N(LP)	N: s(26.91%)p (73.07%)d (0.02%) f (0.01%)
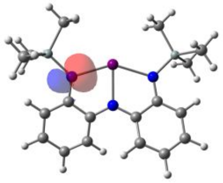	1.72	N(LP)	N: s(26.91%)p (73.07%)d (0.02%) f (0.01%)
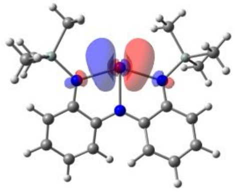	0.36	Bi (LV)	Bi: s(0.00%)p (99.80%)d (0.06%) f (0.14%)

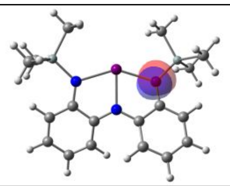	1.58	N(LP)	N: s(0.51%)p(99.44%)d (0.03%) f (0.02%)
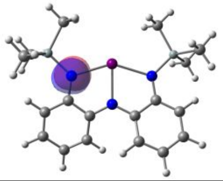	1.58	N(LP)	N: s(0.51%)p(99.44%)d (0.03%) f (0.02%)
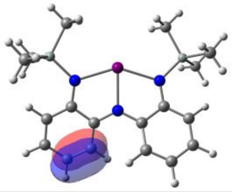	1.74	C(50.01%) – C(49.99%)	C: s(0.00%)p (99.93%)d (0.02%) f (0.04%)<br>C: s(0.00%)p (99.93%)d (0.03% f (0.04%)
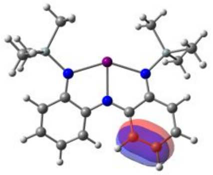	1.74	C(50.01%) – C(49.99%)	C: s(0.00%)p (99.93%)d (0.02%) f (0.04%)<br>C: s(0.00%)p (99.93%)d (0.03% f (0.04%)
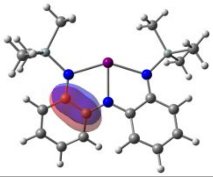	1.51	C(46.61%) – C(53.39%)	C: s(0.00%)p (99.93%)d (0.03%) f (0.04%)<br>C: s(0.00%)p (99.93%)d (0.02%) f (0.05%)
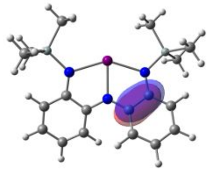	1.51	C(46.61%) – C(53.39%)	C: s(0.00%)p(99.93%)d (0.03%) f(0.04%)<br>C: s(0.00%)p (99.93%)d (0.02%) f(0.05%)
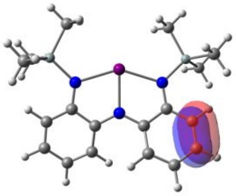	1.75	C(51.92%) – C(48.08%)	C: s(0.01%)p(99.92%)d (0.02%)f (0.04%)<br>C: s(0.02%)p(99.91%)d (0.03%)f (0.04%)
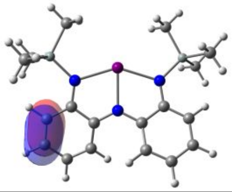	1.75	C(51.92%) – C(48.08%)	C: s(0.01%)p(99.92%)d (0.02%)f (0.04%)<br>C: s(0.02%)p(99.91%)d (0.03%)f (0.04%)

Table S16. Lewis structure-constrained NBO results of **5** at B3LYP-D3(BJ)/def2-TZVPP level of theory. Total non-Lewis (1.80%).

Orbital	Occ	Contribution from atoms to orbitals	Atomic orbitals
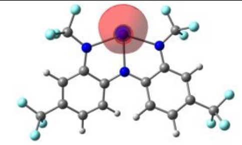	1.99	Bi(LP)	Bi: s(93.58%)p (6.41%)d (0.01%)f(0.00%)
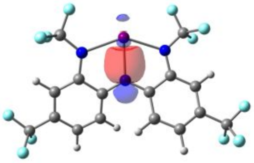	1.95	Bi(15.99%)- N(84.01%)	Bi: s(6.90%)p(92.65%)d (0.18%) f(0.26%)<br>N: s(24.97%)p (75.00%)d (0.02%) f(0.01%)
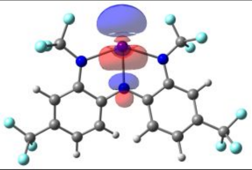	0.11	Bi(84.01%) – N(15.99%)	Bi: s(6.90%)p(92.65%)d (0.18%) f (0.26%)<br>N: s(24.97%)p (75.00%)d (0.02%) f (0.01%)
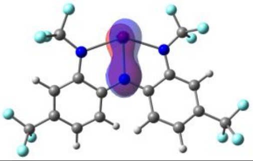	1.78	Bi(23.89%) – N(76.11%)	Bi: s(0.00%)p (99.70%)d (0.16%) f (0.14%)<br>N: s(0.00%)p (99.98%)d (0.00%) f (0.02%)
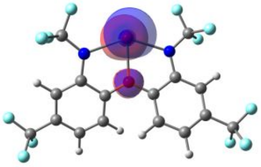	0.62	Bi(76.11%) – N(23.89%)	Bi: s(0.00%)p (99.70%)d (0.16%) f (0.14%)<br>N: s(0.00%)p (99.98%)d (0.00%) f (0.02%)
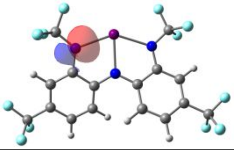	1.73	N(LP)	N: s(33.92%)p (66.05%)d (0.02%)f (0.01%)
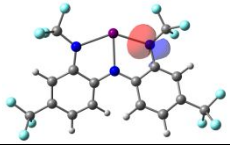	1.73	N(LP)	N: s(33.92%)p (66.05%)d (0.02%) f (0.01%)
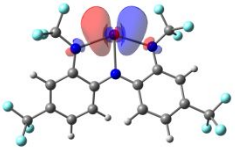	0.35	Bi(LV)	Bi: s(0.00%)p (99.80%)d (0.03%) f (0.17%)
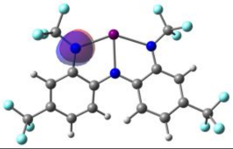	1.60	N(LP)	N: s(0.46%)p(99.48%)d (0.03%) f (0.03%)
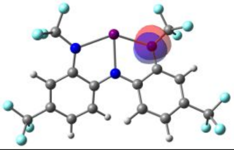	1.60	N(LP)	N: s(0.46%)p(99.48%)d (0.03%) f(0.03%)

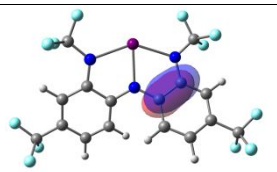	1.53	C(47.87%) – C(52.13%)	C: s(0.00%)p (99.92%)d (0.02%) f (0.05%)<br>C: s(0.01%)p (99.93%)d (0.02%) f (0.05%)
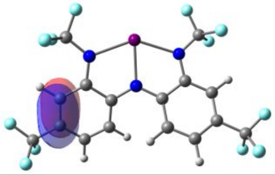	1.73	C(48.71%) –C(51.29%)	C: s(0.01%)p (99.91%)d (0.04%) f (0.05%)<br>C: s(0.03%)p(99.90%)d (0.02%) f (0.05%)
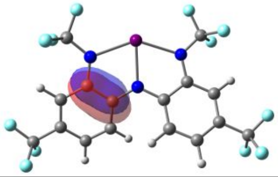	1.53	C(47.87%) – C(52.13%)	C: s(0.00%)p (99.92%)d (0.02%) f(0.05%)<br>C: s(0.01%)p (99.93%)d (0.02%) f(0.05%)
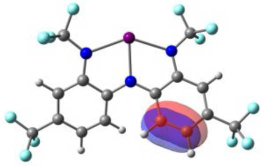	1.73	C(50.84%) – C(49.16%)	C: s(0.00%)p (99.92%)d (0.03%) f(0.04%)<br>C: s(0.00%)p (99.92%)d (0.03%) f (0.05%)
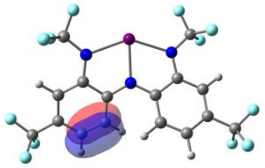	1.73	C(50.84%) – C(49.16%)	C: s(0.00%)p (99.92%)d(0.03%) f(0.04%)<br>C: s(0.00%)p (99.92%)d (0.03%) f (0.05%)
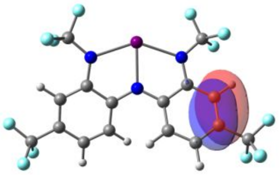	1.73	C(48.71%) – C (51.29%)	C: s(0.01%)p (99.91%)d (0.04%) f (0.05%)<br>C: s(0.03%)p(99.90%)d (0.02%) f(0.05%)

Table S17. Lewis structure-constrained NBO results of **6** at B3LYP-D3(BJ)/def2-TZVPP level of theory. Total non-Lewis (2.06%).

Orbital	Occ	Contribution from atoms to orbitals	Atomic orbitals
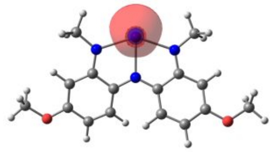	1.99	Bi(LP)	Bi: s(91.83%)p (8.17%)d (0.00%) f (0.00%)
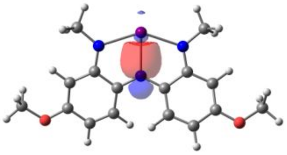	1.95	Bi (16.54%) – N(83.46%)	Bi: s(8.60%)p(90.99%)d (0.17%)f (0.25%)<br>N: s(24.34%)p (75.64%)d (0.02%)f (0.01%)
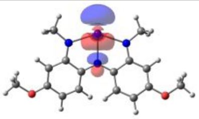	0.15	Bi(83.46%) – N (16.54%)	Bi: s(8.60%)p(90.99%)d (0.17%)f (0.25%)<br>N: s(24.34%)p (75.64%)d (0.02%)f (0.01%)
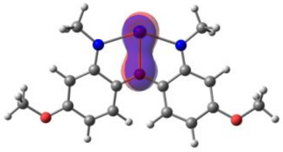	1.80	Bi(32.44%) – N(67.56%)	Bi: s(0.00%)p (99.85%)d (0.06%) f (0.09%)<br>N: s(0.00%)p (99.97%)d (0.00%)f (0.02%)
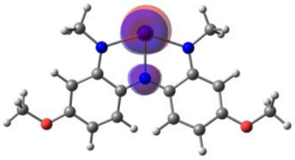	0.80	Bi(67.56%) – N(32.44%)	Bi: s(0.00%)p (99.85%)d (0.06%)f (0.09%)<br>N: s(0.00%)p (99.97%)d (0.00%)f (0.02%)
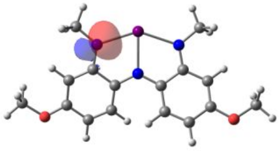	1.71	N(LP)	N: s(33.64%)p (66.33%)d (0.02%) f (0.01%)
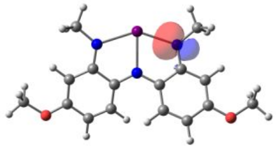	1.71	N(LP)	N: s(33.64%)p (66.33%)d (0.02%) f (0.01%)
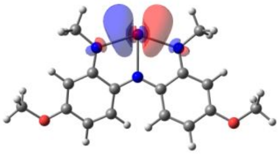	0.37	Bi(LV)	N: s(0.00%)p (99.81%)d (0.03%) f(0.15%)
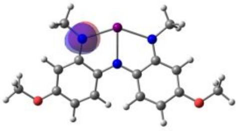	1.54	N(LP)	N: s(0.33%)p(99.62%)d (0.03%) f (0.02%)
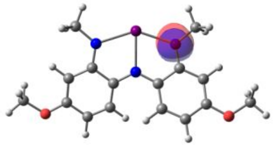	1.54	N(LP)	N: s(0.33%)p(99.62%)d (0.03%) f (0.02%)

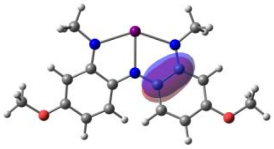	1.50	C(45.11%) – C(54.89%)	C: s(0.00%)p(99.93%)d (0.02%) f (0.05%)<br>C: s(0.01%)p (99.93%)d (0.01%) f (0.05%)
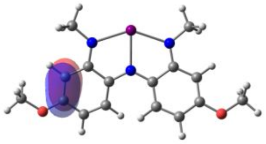	1.76	C(57.11%) – C(42.89%)	C:s(0.01%)p (99.91%)d (0.03%)f (0.05%)<br>C: s(0.01%)p(99.90%)d (0.05%)f (0.04%)
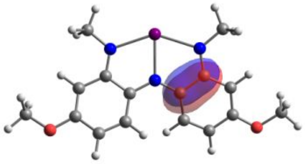	1.50	C(45.11%) – C(54.89%)	C: s(0.00%)p (99.93%)d (0.02%)f (0.05%)<br>C: s(0.01%)p (99.93%)d (0.01%)f (0.05%)
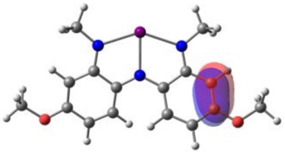	1.76	C(57.11%) –C(42.89%)	C: s(0.01%)p (99.91%)d (0.03%) f(0.05%)<br>C: s(0.01%)p(99.90%)d (0.05%)f(0.04%)
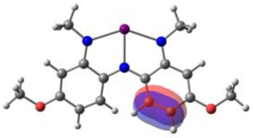	1.77	C(48.24%) – C(51.76%)	C: s(0.00%)p (99.93%)d (0.03%)f (0.04%)<br>C: s(0.00%)p (99.92%)d (0.03%)f (0.05%)
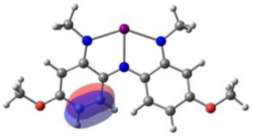	1.77	C(48.24%) – C(51.76%)	C: s(0.00%)p (99.93%)d (0.03%)f (0.04%)<br>C: s(0.00%)p (99.92%)d (0.03%)f (0.05%)

References

1. Soran, A. P.; Silvestru, C.; Breunig, H. J.; Balázs, G.; Green, J. C., Organobismuth(III) Dihalides with T-Shaped Geometry Stabilized by Intramolecular N→Bi Interactions and Related Diorganobismuth(III) Halides. *Organometallics* **2007,** *26* (5), 1196-1203.
2. Soran, A.; Breunig, H. J.; Lippolis, V.; Arca, M.; Silvestru, C., Monoorganobismuth(III) dihalides containing the new pincer 2,6-{MeN(CH2CH2)2NCH2}2C6H3 ligand: solution NMR, vibrational and single-crystal X-ray studies. *Dalton Trans* **2009,** (1), 77-84.
3. Qiu, R.; Yin, S.; Zhang, X.; Xia, J.; Xu, X.; Luo, S., Synthesis and structure of an air-stable cationic organobismuth complex and its use as a highly efficient catalyst for the direct diastereoselective Mannich reaction in water. *Chem Comm* **2009,** (31), 4759-4761.
4. Vránová, I.; Alonso, M.; Lo, R.; Sedlák, R.; Jambor, R.; Růžička, A.; Proft, F. D.; Hobza, P.; Dostál, L., From Dibismuthenes to Three-and Two-Coordinated Bismuthinidenes by Fine Ligand Tuning: Evidence for Aromatic BiC3N Rings through a Combined Experimental and Theoretical Study. *Chem. - Eur. J.* **2015,** *21* (47), 16917-16928.
5. Balasubramaniam, S.; Kumar, S.; Andrews, A. P.; Varghese, B.; Jemmis, E. D.; Venugopal, A., A dicationic bismuth (III) Lewis acid: catalytic hydrosilylation of olefins. *European Journal of Inorganic Chemistry* **2019,** *2019* (28), 3265-3269.
6. Ramler, J.; Krummenacher, I.; Lichtenberg, C., Bismuth Compounds in Radical Catalysis: Transition Metal Bismuthanes Facilitate Thermally Induced Cycloisomerizations. *Angew Chem Int Ed Engl* **2019,** *58* (37), 12924-12929.
7. Mokrai, R.; Barrett, J.; Apperley, D. C.; Batsanov, A. S.; Benko, Z.; Heift, D., Weak Pnictogen Bond with Bismuth: Experimental Evidence Based on Bi-P Through-Space Coupling. *Chemistry* **2019,** *25* (16), 4017-4024.
8. Soran, A. P.; Silvestru, C.; Breunig, H. J.; Balázs, G.; Green, J. C., Organobismuth (III) dihalides with T-shaped geometry stabilized by intramolecular N→ Bi interactions and related diorganobismuth (III) halides. *Organometallics* **2007,** *26* (5), 1196-1203.
9. Šimon, P.; Jambor, R.; Růžička, A.; Dostál, L., Oxidative Addition of Diphenyldichalcogenides PhEEPh (E = S, Se, Te) to Low-Valent CN- and NCN-Chelated Organoantimony and Organobismuth Compounds. *Organometallics* **2013,** *32* (1), 239-248.
